# Mutual (Mis)understanding: Reframing Autistic Pragmatic “Impairments” Using Relevance Theory

**DOI:** 10.3389/fpsyg.2021.616664

**Published:** 2021-04-29

**Authors:** Gemma L. Williams, Tim Wharton, Caroline Jagoe

**Affiliations:** ^1^School of Humanities, University of Brighton, Brighton, United Kingdom; ^2^School of Linguistic, Speech and Communication Sciences, Trinity College Dublin, University of Dublin, Dublin, Ireland

**Keywords:** autism, intersubjectivity, relevance theory, communication, double empathy problem

## Abstract

A central diagnostic and anecdotal feature of **autism** is difficulty with social **communication**. We take the position that communication is a two-way, **intersubjective** phenomenon—as described by the **double empathy problem**—and offer up **relevance theory** (a cognitive account of utterance interpretation) as a means of explaining such communication difficulties. Based on a set of proposed heuristics for successful and rapid interpretation of intended meaning, relevance theory positions communication as contingent on shared—and, importantly, **mutually** recognized—“relevance.” Given that autistic and non-autistic people may have sometimes markedly different embodied experiences of the world, we argue that what is most salient to each interlocutor may be mismatched. Relevance theory would predict that where this salient information is not (mutually) recognized or adjusted for, mutual understanding may be more effortful to achieve. This paper presents the findings from a small-scale, linguistic ethnographic study of autistic communication featuring eight core autistic participants. Each core autistic participant engaged in three naturalistic conversations around the topic of loneliness with: (1) a familiar, chosen conversation partner; (2) a non-autistic stranger and (3) an autistic stranger. Relevance theory is utilized as a frame for the linguistic analysis of the interactions. Mutual understanding was unexpectedly high across all types of conversation pairings. In conversations involving two autistic participants, flow, rapport and intersubjective attunement were significantly increased and in three instances, autistic interlocutors appeared to experience improvements in their individual communicative competence contrasted with their other conversations. The findings have the potential to guide future thinking about how, in practical terms, communication between autistic and non-autistic people in both personal and public settings might be improved.

## Introduction

Issues around autistic communication were identified as a top priority for autism research by stakeholders in an independent James Lind Alliance Priority Setting Partnership priority-setting report (Cusack and Sterry, 2016, p. 6). Community priority-setting is an important means of ensuring that research aligns with the needs of stakeholders: something that is essential if we want outcomes to be genuinely meaningful (Milton and Bracher, 2013; Chown et al., 2017). Yet, while language and communication in autism is clearly a key area for research, it remains something of a “blind spot” (De Jaegher, 2013, p. 14; Morrison et al., 2019b): this study addresses this issue. Using a small corpus of transcribed, naturalistic conversations involving eight core autistic adult participants across three different conversation conditions, it explores how implicit expectations of shared relevance contribute to breakdowns in understanding between autistic and non-autistic interlocutors^1^.

## Research Context

### Autism

The past three decades have seen interest in autism as a field of research boom [Interagency Autism Coordinating Committee (IACC), 2013; Pellicano, 2014], coinciding with a dramatic shift in terms of how autism is defined (Happé and Frith, 2020). Medically, autism is classified as a neurodevelopmental disorder, hanging on a set of observed and reported behavioral characteristics. These characteristics, largely based on Wing and Gould's “Triad of Impairments” (Wing and Gould, 1979), are described as impairments in social interaction, in (social) imagination (i.e., demonstrating restricted interests and repeated or stereotyped behaviors) and in communication (see DSM-5 criteria, American Psychiatric Association, 2013). Communication, for these diagnostic purposes, “refers to the full range of both verbal/linguistic and non-verbal (including gesture and intonation) means for interacting with others” (Tager-Flusberg, 1999, p. 325).

Autism is also now commonly conceptualized as a form of neurodivergence i.e., “a specific neurological state” (Beardon, 2017, p. 13) or “disposition” (Milton, 2014) that is “different, not less” (Fletcher-Watson and Happé, 2019, p. 23). The study reported on below adopts this perspective. While the shifting parameters and difficulty in identifying a specific biological cause have led to consternation about the validity of the construct that is autism (e.g., Cushing, 2013; Verhoeff, 2013; Timimi and McCabe, 2016), others have argued that the term is nonetheless useful for those whose lived experiences it describes (e.g., Milton, in Milton and Timimi, 2016; Beardon, 2017; Woods et al., 2018; Chapman, 2020).

Based on original findings by Baron-Cohen et al. (1985) and numerous replication studies, autism research has long been characterized by the belief that impaired theory of mind is a defining trait. However, in addition to the recent evidence demonstrating non-autistic people's inability to accurately impute the mental states of autistic people (see below section Autistic Communication and the “Double Empathy Problem”), the idea that non-autistic children and adults consistently perform at ceiling level in ToM tasks has also now been challenged (e.g., see Samson and Apperly, 2010; Warnell and Redcay, 2019). Furthermore, Peterson and Wellman (2019) discovered that autistic children follow a complete, but atypical sequence of ToM stage progression. At the sequential stage when typically developing children are acquiring the ability to represent false beliefs, autistic children are instead developing the ability to understand that underlying emotions can be hidden. It is possible that the over-reliance on false belief test measures in early childhood has skewed our appreciation for the potential of ToM development in autism.

#### Autistic Communication and the “Double Empathy Problem”

Over the past two decades, research into autistic sociality and communication has begun to turn its gaze toward *intersubjectivity*. Taking a phenomenological perspective, intersubjectivity acknowledges that as embodied social agents we share in some degree of a “co-conception or co-orientation to the world” (Schegloff, 1992, p. 1296). Intersubjectivity functions as a counter to a solipsistic view whereby the individual mind has primacy and emphasizes the inter-relational aspect of selves and selfhood.

Communication, viewed intersubjectively, does not occur in a void, nor solely in the mind of one individual: it is a social and interactive phenomenon. In order to reflect this, and in opposition to traditional explanations of autism that have situated the mind-reading “failures” assumed central to pragmatic breakdown in the minds/brains of the autistic individuals, Milton (2012) proposes the DEP. This holds that cross-dispositional communication (i.e., between two speakers of different neurotypes) is troubled by “a disjuncture in reciprocity between two differently disposed social actors” (Milton, 2012, p. 884), “who hold different norms and expectations of each other” (Milton et al., 2018, p. 1). Misunderstanding or lack of understanding is not a consequence of autistic “impairment” but a mutual failure in reaching consensus through bidirectional empathy.

Recent empirical autism research, situated largely in the social sciences, has begun to provide evidence in support of the DEP and illuminate the difficulties non-autistic people also experience in understanding autistic people: such as difficulty in inferring autistic affective and mental states (Brewer et al., 2016; Edey et al., 2016; Sheppard et al., 2016; Heasman and Gillespie, 2017; Hubbard et al., 2017) and a tendency toward negative thin slice judgements about autistic people (Sasson et al., 2017; Morrison et al., 2019a). Research has also highlighted how autistic people can demonstrate highly successful and nuanced socio-communicative abilities when among others of a similar neurotype (Crompton et al., 2019a,b; Heasman and Gillespie, 2019; Morrison et al., 2019b).

Linguistic ethnographic research (such as that by Ochs and Solomon, 2010; Sirota, 2010; Sterponi and Fasulo, 2010) as well some other work on autistic communication (e.g., Bogdashina, 2005; Chown, 2012; De Jaegher, 2013, 2020; Sterponi and de Kirby, 2016; Di Paolo et al., 2018), has led the way in taking an intersubjective approach to autism and autistic language use. Autistic participants are approached as situated, interactive agents within their familiar worlds, and from “a phenomenological, rather than a biomedical, point of view” (Solomon and Bagatell, 2010, p. 2).

Linguistic analyses that begin with the premise of asking “what is this utterance doing?,” instead of automatically problematizing it, can uncover previously overlooked competences. Sterponi and de Kirby (2016) demonstrate that some of the key characteristics of so-called “impaired” autistic language—pronoun atypicality, echolalia and pragmatically atypical utterances—are revealed to have potentially alternative explanations, such as echolalia functioning as a form of perspective-taking. While these studies explore new territory in the analysis of autistic language use, many involve child-adult dyads which are necessarily asymmetric. The present study aims to apply this same approach to an analysis of adult autistic language use.

#### Monotropism

*Monotropism* (Murray et al., 2005; Murray, 2018, 2020) is a compelling interest-based account of autism, based within a dynamic, ecological, model of minds. However, it has received little mainstream attention since its conception 15 years ago. Originally proposed by three autistic scholars, the theory begins from the position that the mind is, essentially, an interest system—a starting place not dissimilar to that of the weak central coherence theory—and that “atypical strategies for the allocation of attention” (Murray et al., 2005, p. 139) are the central cause of the various autistic social and behavioral manifestations. Murray et al. propose that the degree or breadth of attention allocation in humans is “normally distributed” and (largely) “genetically determined” (Murray et al., 2005, p. 140), with some people possessing a greater tendency toward multiply focused attention (*polytropism)*, and others a tendency toward more narrowly focused attention (monotropism). Those identified or identifying as autistic will find themselves at the far end of this distribution with a highly narrow “attention tunnel.” Where polytropic minds comfortably entertain many simultaneous interests, each moderately aroused, the monotropic mind will maintain only very few simultaneous interests, with each one highly aroused and intensely focused upon.

The monotropic account offers a unified explanation for the many different features associated with autism. The restricted and repetitive behaviors and interests (see DSM-5 criteria, American Psychiatric Association, 2013) can be explained by attention firing into “monotropic superdrive” (Murray et al., 2005, p. 143) and entraining itself onto one self-pleasing task or topic. Crucially, social and communicative difficulties may come about as a consequence of a difficulty in processing, at speed, information from a variety of simultaneous channels (audio, visual, socio-cultural encyclopedic knowledge, etc…); a skill better suited to polytropic individuals with less narrowly and intensely focused attention. Similarly, stimuli outside of the monotropic attention tunnel may carry reduced salience, a potential difficulty when communication is considered in relevance theoretic terms.

### Relevance Theory and Mutual Manifestness

Building on Grice's (1975) inferential model of communication, relevance theory (Sperber and Wilson, 1986, 1995) regards communication as involving more than the simple encoding and decoding of a linguistically encoded meaning. Intended meanings are retrieved *via* a context-bound search for optimal relevance, where “relevance” is defined as a balance between the greatest number of communicative effects achieved for the lowest amount of processing effort. The approach is underpinned by two principles. The Cognitive Principle of Relevance holds that the search for relevance is a central goal of human cognition: this is a claim that is backed up by work in cognitive science^2^. The Communicative Principle of Relevance takes it that because human cognition is geared to the search for relevance, speakers ensure that their utterances come with a presumption of their own optimal relevance. Hearers can therefore safely assume that the utterance is relevant enough to merit the effort required to process it. In this way, speakers, therefore, can ensure hearers will pay attention to them.

This mutual calibration of shared cognitive space is central to relevance theory's notion of ostensive-inferential communication. All facts and assumptions both actually and *potentially* available to any individual as a result of interaction between their physical environment and their cognitive abilities are considered “manifest” to them (Sperber and Wilson, 1986, 1995). The set of assumptions that is manifest to an individual at any given time constitutes their “cognitive environment,” and two people who share assumptions are said to share a cognitive environment. Finally, any shared cognitive environment in which it is manifest which people share it is described as a “mutual cognitive environment.” As Sperber and Wilson put it (1986; 1995, p. 42): “[I]n a mutual cognitive environment, every manifest assumption is… mutually manifest.” Mutual manifestness is the basis from which judgements relating to the optimal relevance of an utterance are formed.

For communication to work, meta-representational abilities that enable a speaker or listener to infer what their interlocutor has in mind, and what their interlocutor should reasonably believe them *they* have in mind, are essential. For this reason, relevance theory has largely been used to explain the cognitive mechanisms of (both successful and unsuccessful) utterance interpretation in typically-developed communicators with typical ToM abilities. Of the few studies that have applied a relevance theoretic lens to autistic communication (Happé, 1991, 1993, 1995; Leinonen and Kerbel, 1999; Papp, 2006; Loukusa et al., 2007; Leinonen and Ryder, 2008; Wearing, 2010), all have approached the matter from the perspective that autistic people have impaired ToM abilities^3^. Autistic participants have been used as case studies to validate relevance theory's claims on the mechanisms of utterance interpretation.

We suggest that because of their divergent sensory and perceptual experiences (Bogdashina, 2010; De Jaegher, 2013; Beardon, 2017), and markedly different patterns of attention (Murray et al., 2005), it is plausible that autistic people attribute relevance in significantly different ways to non-autistic people. What is and is not relevant, which facts and assumptions are manifest at any given time, and the way in which representations are organized and accessible, may be more markedly different than those of a non-autistic interlocutor, or indeed, a different autistic interlocutor. The degree of cognitive effort required to generate certain cognitive effects will therefore also be different. We argue that both autistic and non-autistic speakers communicate according to the principles of relevance theory. We suggest that it is where assumptions of mutual manifestness are erroneously made (by either or both parties), that mutual understanding will break down. In this way we resituate the responsibility of breakdowns in understanding on the shoulders of all parties involved, as relevance theory has always intended. This position accords with theories that posit that humans are most successful at inferring the mental and affective states of those others who are most cognitively similar to themselves, and that interactions between autistic and non-autistic people are prime examples of where such conditions are infelicitous (De Jaegher, 2013, 2020; Bolis et al., 2017; Fein, 2018; Chapman, 2019; Conway et al., 2019a,b).

## Materials and Methods

### Aims

This study took the form of a small-scale linguistic-ethnographic case-study featuring eight core autistic participants. The primary aim of this study was to investigate the strength of the proposal that the relevance theoretic notion of mutual manifestness might serve to support the DEP-based theory of mutual misunderstanding in cross-dispositional communication, based on an expectation that in such circumstances both interlocutors may be inclined to make faulty assumptions of mutual manifestness.

### Participant Selection and Design

Eight core autistic participants were recruited through Assert, a local autism support charity acting as gatekeeper, and invited to take part in three naturalistic conversations of roughly 10 min each. Assert is a member led organization, founded in 2002, that supports autistic people traditionally identified as being “high functioning,” or having Asperger's Syndrome, along with their family members, partners or carers. The conversations were focused around the loose topic of loneliness. We wanted to strike the balance between providing some form of framework for the conversations, not unduly directing or influencing their structure, and maintaining a degree of parity across the conversation conditions.

Since there is a “a lost generation of people who were previously excluded from a diagnosis” (Lai and Baron-Cohen, 2015, p. 1013), and achieving a diagnosis of autism in adulthood is not easy (Taylor and Marrable, 2011), we decided that stipulating a formal autism diagnosis seemed unnecessarily limiting. Instead, autistic participants were asked about their autism diagnosis at recruitment and again within their consent forms. All autistic participants reported a diagnosis of either “autism level 1,” “autism spectrum condition,” or “Asperger's syndrome:” the various terminology reflecting the differing times at which they received their diagnoses

The sampling in this study was purposeful (Patton, 1999; Palinkas et al., 2015); core autistic participants were selected on account of their being autistic adults who used language as their primary mode of communication as well as their availability and willingness to engage with the research. Within these parameters, we chose to not impose or collect any further demographic stipulations, so as to allow for as much variability as possible. Finding a group of “typical” autistic people is nigh impossible, given the characteristic heterogeneity of autism (e.g., see Beardon, 2017; Fletcher-Watson and Happé, 2019).

Non-autistic participants were asked both at recruitment and within their consent form to confirm that they did not have a history of speech and language difficulties, autism or learning difficulties. Non-familiar stranger participants had been invited to take part in a Linguistics PhD research project looking at communication across pairs of strangers, with no mention made at any stage that their interlocutors would be autistic. The familiar, chosen conversation partners were not asked about an autism diagnosis although in all but two cases they identified themselves as non-autistic, with one chosen partner (Participant code X6) not mentioning it either way, and another (Participant code X3) identifying herself as autistic. The only important criterion for the chosen, familiar participants was the strength of familiarity they had with the core autistic participants.

### Making the Experience Meaningful

In order to obtain naturalistic data, it was important to generate and facilitate conversations that were not in any way contrived. In addition, in making the data-collecting activity meaningful in its own right, the research project could become a mutually beneficial endeavor to both us as researchers and to the participants: a cornerstone of participatory and community-based research (Milton and Bracher, 2013; Chown et al., 2017; Elson et al., 2018; Fletcher-Watson et al., 2019).

Loneliness is a “universal affliction” (McGraw, 1995, p. 43) that can not only cause significant distress but also functions as a risk factor for various health problems and increased mortality rates (Holt-Lunstad et al., 2010; Valtorta et al., 2016). Autistic people are especially prone to loneliness and social isolation (National Autistic Society, 2018), further associated with increased depression and anxiety (Mazurek, 2014) and self-harm (Hedley et al., 2018). Given that was potentially relevant to the participants, we chose loneliness in Brighton and Hove as the central focus of the conversations (see Williams, 2020)^4^.

**Table 1:** 

****	**Core autistic participant**		**Conversation condition/configuration**	**Interlocutor**	
	**Code**	**Demographic details**		**Code**	**Demographic details**
Suite 1	A1	Autistic male with additional learning difficulties, in his 50s	^*^Cross-dispositional (familiar)	X1	Male work colleague
			^*^Cross-dispositional (unfamiliar)	B1	Non-autistic stranger, male, early 20s
			^*^Matched-dispositional (unfamiliar)	A2	Autistic female, mid 30s–mid 40s
	A2	Autistic female, in her mid 30s–mid 40s	Cross dispositional (familiar)	X2	Male friend of A2
			Cross-dispositional (unfamiliar)	B1	Non-autistic stranger, early 20s
Suite 2	A3	Autistic female, French-English bilingual, in her 50s	^*^Matched-dispositional (familiar)	X3	Autistic female friend of A3's, in her 50s
			Cross-dispositional (unfamiliar)	B2	Female non-autistic stranger, early 20s
			^*^Matched-dispositional (unfamiliar)	A4	Autistic male, in his 50s
	A4	Autistic male, in his 50s	^*^Cross dispositional (familiar)	X4	A4's non-autistic wife, 50s
			^*^Cross-dispositional (unfamiliar)	B3	Female non-autistic stranger, mid 20s
Suite 3	A5	Autistic female, in her mid 30s−40s	Cross dispositional (familiar)	X5	Female Assert staff member, 30s
			^*^Cross-dispositional (unfamiliar)	B4	Female non-autistic stranger, 30s
			^*^Matched-dispositional (unfamiliar)	A6	Autistic female, in her 30s
	A6	Autistic female, in her 30s	^*^Cross dispositional (familiar)	X6	Female friend of A6
			^*^Cross-dispositional (unfamiliar)	B4	Female non-autistic stranger, 30s
Suite 4	A7	Autistic female, in her early-mid 20s	^*^Cross dispositional (familiar)	X7	Older sister of A7, late 20s/early 30s
			Cross dispositional (unfamiliar)	B5	Female non-autistic stranger, late 40s
			^*^Matched-dispositional (unfamiliar)	A8	Autistic male, in his 40s
	A8	Autistic male, in his 40s	^*^Cross dispositional (familiar)	X8	Female non-autistic housemate and friend of A8
			^*^Cross-dispositional (unfamiliar)	B6	Male non-autistic stranger, early 20s

* *Extracts from these conversations are used as illustrative extracts for the purposes of this paper*.

### Procedure

Five sessions were scheduled at different times over 3 days in order to make the “Talking Together” project accessible to as many people as possible. In each session, a series of five conversations took place; (1) a core autistic participant (A) with their chosen partner (X); (2) a further A with their chosen X; (3) both core As together; (4) the first A with an unfamiliar, non-autistic participant (B); and (5) the second A with a B participant. The conversations were scheduled for every 20 min, meaning that each core A participant only had one 20-min wait between conversations. Conversations took place in a small private meeting room at the Assert premises in the center of Brighton, just along from the communal waiting room where participants and their familiar partners could wait, talk, rest and have refreshments.

For each of the three conversation pairings, a (different) set of two prompt questions (see Supplementary Material) were provided in order to give the participants somewhere to begin, although it was explained that the questions were just there as a guide. Prompts were designed to elicit personal experiences of loneliness, thoughts about loneliness in Brighton and Hove more specifically and to invite ideas around how address those problems within the city.

Conversations were digitally recorded and professionally transcribed according to the transcription conventions adopted for use in Conversation Analysis (originally developed by Jefferson, 1984, see Supplementary Material) to include information pertaining to pauses, word stress, and intonation etc., whilst remaining readable.

### Data Analysis

Relevance theory is not a methodology but a cognitive theory of utterance interpretation. There is, however, precedent for the application of a relevance theoretic lens to the analysis of conversational data (e.g., Leinonen and Kerbel, 1999; Jagoe, 2012, 2015; Jagoe and Smith, 2016; Jagoe and Wharton, 2021). Jagoe (2015), for example, analyzed the delusional talk of seven individuals with schizophrenia engaged in conversation with the researcher (a speech and language therapist) from a relevance theoretic perspective^5^. Relevance theory provided the theoretical descriptive basis for human communication, on which the analysis was built. Furthermore, it served there as the explanatory and theoretical framework underpinning interpretation of the data, with the notion of mutual manifestness (or the lack thereof) functioning “as a useful construct with which to understand the to-and-fro of the meaning negotiation process” (Jagoe, 2015, p. 66). In Leinonen and Kerbel's (1999) relevance theoretic analysis of the talk-in-interaction of three children with pragmatic impairments, transcripts were scanned for “instances of communicative “oddness,” created either by the children or the adults” (Leinonen and Kerbel, 1999, p. 372). Approaching the analysis of the data from the theoretical basis of relevance theory, combined with an open-minded, inquisitive attitude and asking “why that, now?” (Sterponi and de Kirby, 2016, p. 398) should, in principle, afford a grounded, reliable yet sensitive reading of the data.

#### Data Analytic Method

The study presented in this paper uses qualitative methods, situated in an interpretative paradigm. Qualitative coding and analysis is an iterative, reflexive process (Braun and Clarke, 2006, 2020; Tracy, 2010) that develops over an extended period of time. According to Braun and Clarke (2020, p. 6, 7), such inductive and reflexive approaches “fully embrace qualitative research values and the subjective skills the researcher brings to the process.” In our case, the analysis took place over a period of months in conversation between the three authors. The primary analysis was undertaken by the lead author (GW—whose doctoral research this research represented) with ongoing supervision, discussion and reviewing of coding, extract selection and analysis provided by the two further co-authors. This triangulation of analytic perspectives, we feel, was further strengthened by our combined diversity of dispositions (two of us are non-autistic and one of us is autistic). The analysts were not blinded as to the autistic “status” of the interlocutors as this would not have aligned with our linguistic ethnographic approach.

In the initial stage of the analysis, the transcripts were read through several times each in order to become familiar with the form and content of the conversations and the individual interlocutors. These first readings were undertaken within the Nvivo data analysis programme (QSR International Pty Ltd, 2020): software designed to assist in the management of qualitative datasets. Some initial codes were made representing emergent themes relating to the loneliness qualitative content, and stored for the planned secondary analysis to be completed later. In those cases where conversational characteristics were already becoming apparent, these were recorded as notes in the research log.

In the second phase of readings, now focused on the primary research aim, printed transcripts were read through, searching specifically for moments of communication breakdown with the view to analyze them through the lens of mutual manifestness. However, it became quickly evident that there were, in fact, very few instances of communication breakdown through the whole 240 min of transcribed conversational data. If anything, these conversations were consistently characterized by sustained mutual understanding. Further discussion of this surprising finding is provided in section Discussion.

The plan was revised to focus instead on the qualitative differences across the different conversational conditions that had become apparent during the note-taking stage in the first readings. Fresh readings were undertaken of the transcripts, this time adopting a “first person perspective” in order or to “bracket out the researcher's own perspectives and assumptions” (Watts, 2014, p. 4). Detailed notes were made on each conversation, capturing observations, impressions, qualities, and patterns. Coding schemes were developed iteratively, guided by the emergent patterns in the data (see Supplementary Material). The codes were then organized into four inductively-derived “motifs” (N.B not “themes” as these usually refer to qualitative thematic content): “*flow,” “tuning in,” “running along the edges of meaning*,” and “*mutual manifestness*.”

The *flow* motif relates to instances where conversational progressivity was notably fluid or stilted, as marked by characteristics such as “high-quality turn-taking, short response latencies, and few interruptions” (Koudenburg et al., 2017, p. 51); or pauses (within turns), gaps (between turns) and lapses (between sequences), interruptions and long (monologic) turns.

The *tuning-in motif* brings together characteristics of the conversational form and non-propositional content that indicate that interlocutors are “on the same wavelength” (Koudenburg et al., 2017, p. 53). Features of coordination, such as mirroring the other's speech (either by echoing specific words or phrases or offering parallel anecdotes), and finishing the other's sentences combine with evidence of rapport and the presence of shared jokes and humor (a form of affective coordination: Nelson et al., 2016) to create a sense of dyadic synchrony, or “closely aligned intersubjectivity” (Heasman and Gillespie, 2019, p. 916) that Koudenburg et al. (2017) have termed “emergent we-ness” or “solidarity.”

The *running along the edges of meaning* motif borrows its title from an observation made by Sterponi and Fasulo (2010) in their linguistic ethnographic analysis of a young autistic boy (“Aaron”) and his mother engaging in verbal play together. Rather than ignoring Aaron's seemingly meaningless utterance playing with the sound of the word “bug,” she joins him, echoing his utterances until the sequence develops into a joyful, rhymical duet. “Language [use] is set free and allowed to run along the very edges of meaning” (Sterponi and Fasulo, 2010, p. 135).

There were not many instances of linguistic freestyling, but there were moments of left-field, non-tangential topic development and abrupt topic changes—which echoed the low demand for coherence noted in autistic group interactions by Heasman and Gillespie's (2019)—as well as non-words and word play such onomatopoeia etc. These features all seemed to have in common something of the diverging from ordinary, expected discourse and as such were grouped together under the *running along the edges of meaning* motif.

The smaller *mutual manifestness* motif relates to instances where its presence or absence was clear.

For the final stage of the analysis, the transcripts were analyzed once more: this time from a “third person perspective” (i.e., applying “the analyst's thoroughgoing knowledge of a relevant theoretical and*/*or substantive literature,” Watts, 2014, p. 4). From this stance, extracts that might support, qualify, question or contradict existing literature and the hypotheses driving this study were carefully, purposefully selected and are included below.

#### Ethical Approval

This study was granted ethical approval by the Tier II Arts and Humanities Ethics Panel at the University of Brighton. All participants were provided with information sheets at recruitment and again on the day of the research and all gave their written, informed consent in accordance with the Declaration of Helsinki. Information sheets were designed with accessibility for autistic people in mind, drawing on GW's personal autistic insights and advice given in the Participatory Autism Research Starter Pack (Pellicano et al., 2017).

## Results

The conversations contained very few instances of non-understanding. However, what *was* evident, were discernible qualitative differences between those conversations held by cross-dispositional pairs (i.e., A + X; A + B) and those by the exclusively autistic dyads (i.e., A + A). The codes and resulting *motifs* were developed as a means of trying to capture this difference.

Conversations are presented below in four suites of five (e.g., Suite One includes: **A1** + X1 — familiar cross-dispositional condition; **A1** + B1 — unfamiliar cross-dispositional condition; **A1** + *A2* — unfamiliar matched-dispositional condition; *A2* + X2 — familiar cross dispositional condition; *A2* + B1 — unfamiliar cross-dispositional), so as to allow closer comparison between the three conversations of each core “A” participant. Within each suite, extracts are presented where they are relevant to the primary *motifs* in the following order: (1) *flow*; (2) *tuning-in*; and (3) *running along the edges of meaning*. The first and second *motifs* are closely related to one another and so some extracts may, at times, represent both. For that reason, *flow* and *tuning in* are considered together for each suite. For some suites there may not be extracts representing all three *motifs*. Extracts belonging to the final, smaller *motif* of “mutual manifestness” are woven throughout each suite where appropriate.

Transcripts were organized so that the left column represents the speech of the core autistic participant (A) and the right column their interlocutor (X, B, or another A). Where two As are talking, the As are presented in numerical order (e.g., in Conversation 3, A1 is to the left and A2 is to the right). For readers educated in Western traditions, top-to-bottom and left-to-right biases play a part in how the visually recorded spoken word is engaged with (Ochs, 1979). We wanted to center the voice of the voices of the core autistic participants, even if implicitly.

### Suite One

#### Suite One *Flow* and *Tuning in*

Monologic turns were common in this first suite of conversations. In the cross-dispositional conversation between A1 and X1, A1 appears to stumble over constructing his turns. His speech is peppered with fillers, pauses, stuttered words, and rephrases which means that it takes him extra time to arrive at his intended points.


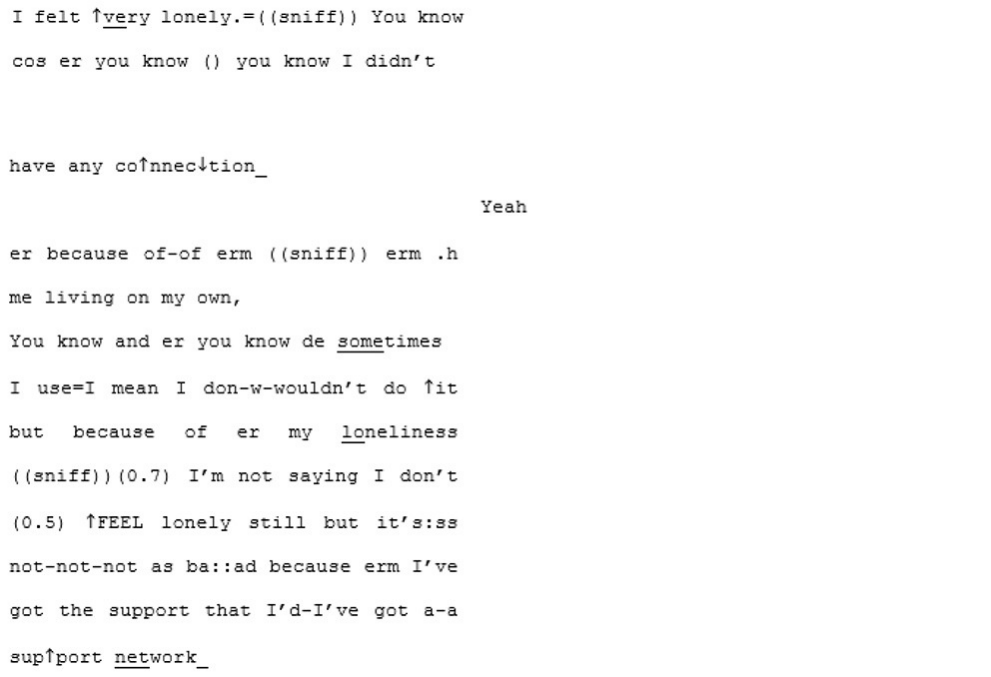


X1, a work colleague of A1's who agreed to come along and participate, is familiar with A1 and appears patient with these long, sometimes labored turns, creating a conversation where A1 has room to speak, but one that has the feel of being lopsided.

In the cross-dispositional conversation between A2 and her familiar interlocutor, X2, there appears to be a greater sense of balance in terms of turn-taking and contributions, but the turns are still often very long (one turn, for example, lasts 45 lines/1 min and 22 s). Again, there are a lot of pauses and gaps, particularly in the first few minutes, and episodes of parallel dialogues where both acknowledge the other's contributions but continue with their own separate topic when the turn passes back to them. Despite A2 introducing X2 as her friend, and them appearing to have a good understanding of each other's day-to-day, the dialogue comes across as rather staid. The conversation remains on a theoretical, intellectual level about the nature and causes of loneliness with not one moment of laughter, enthusiasm, or signal of affect throughout.

In contrast to this is the matched-dispositional conversation, where A1 and A2 meet. Immediately, the conversation has a sense of flow, which continues throughout the interaction. Within moments of beginning their conversation together, A2 correctly predicts what A1 is aiming for, and helps him get there:


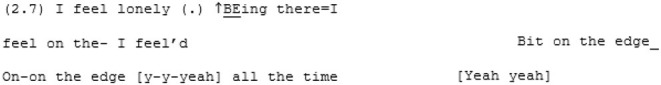


Rather than the parallel dialogues of the previous conversation, this one is characterized by a coherent progression of adjacent turns. Where both A1 and A2—most likely for different reasons—had tended toward long turns across the cross-dispositional conversations (familiar and unfamiliar conditions), here they fall into a fluid rhythm of shorter, responsive turn-taking.

Genuine rapport appears to build too, demonstrated by the mirroring of anecdotes and enthusiastic mutual agreement. In the familiar cross-dispositional condition, A2 sat back when her interlocutor (X2) spoke, giving only minimal backchannel cues (“mmm;” “yeah”). Here she seems more engaged, making contributions that could be understood as enthusiastic, further indicating rapport:


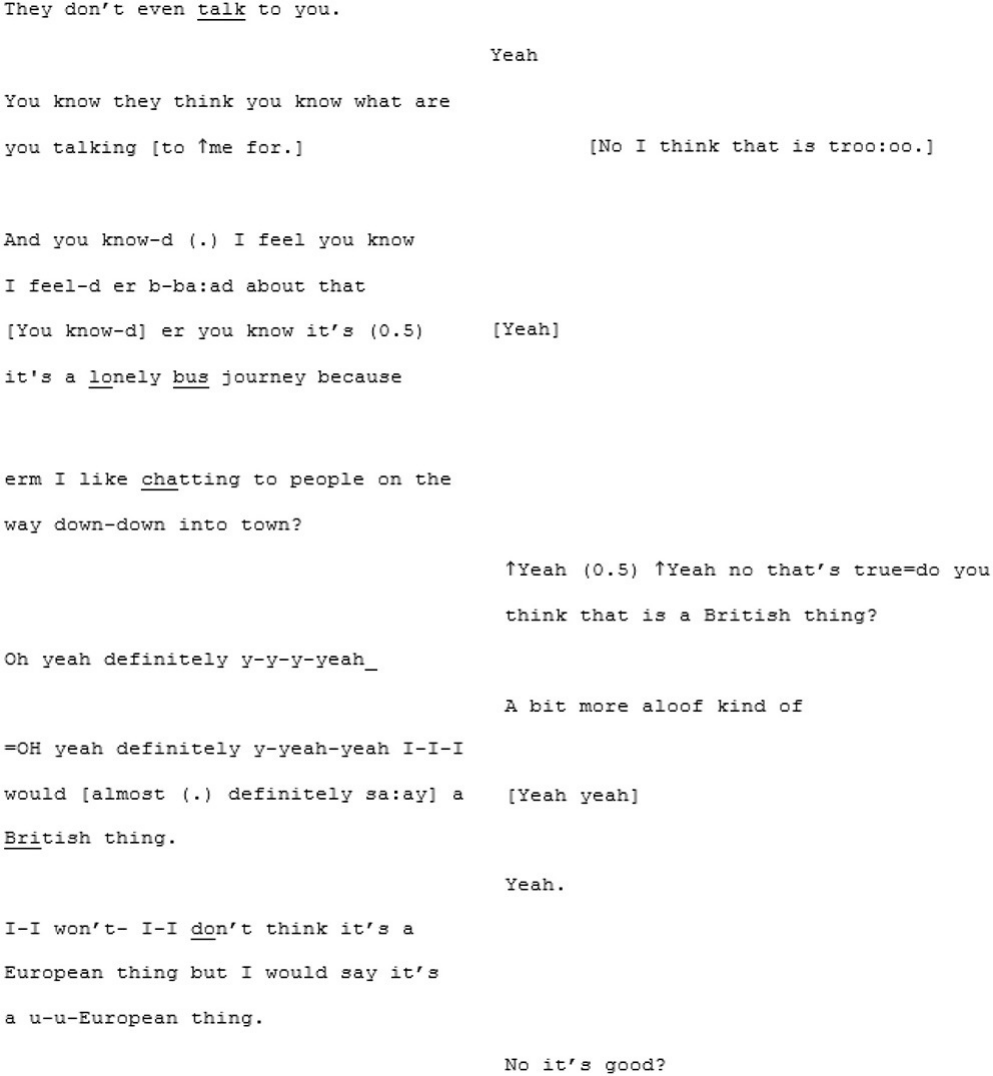


The shared enthusiasm crescendos around lines 52–101, where they discover they both have dogs. A1's dog is clearly a significant and supportive character in A1's life: he is mentioned in all three of his recorded conversations and also during informal discussions in the waiting room. In this matched-dispositional conversation, mention of the dog appears to spark a long sequence full of laughter, emphatic agreement (e.g., “Me too”- line 56; “YEAH tha-tha-that's why that's exactly what I do”—lines 69–70), shared parallel anecdotes and echoic mirroring of the phrase “love… to bits:”


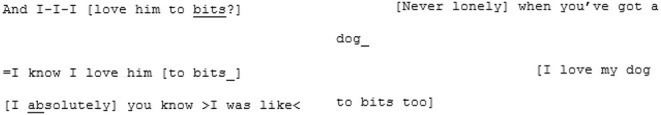


The same topic is seemingly met with limited engagement in both of the cross-dispositional interactions. In A1's interaction with his familiar conversation partner the reference to his dog is something of a non-event, although it could be that the dog is already known to his interlocutor (X1) and its mention not especially newsworthy. However, when his pet is introduced to B1—a (non-autistic) stranger to A1—there also appears to be a distinct lack of engagement:


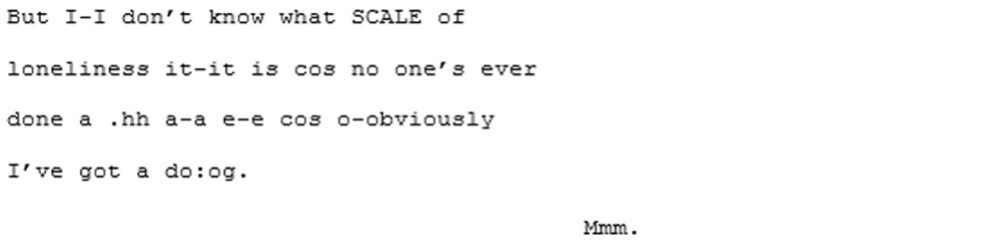


The focus on the topic of his pet could be framed as evidence of one of the diagnostic features of autism: the presence of “highly restricted, fixated interests” (DSM-5, American Psychiatric Association, 2013). Monotropism theorists, however, have long reframed these intense absorptions—sometimes manifesting as encyclopedic knowledge of a specific subject—as highly aroused interests within a monotropic attention tunnel rather than a cognitive deficit (Murray et al., 2005). In an ethnographic study exploring social interactions at an autistic-separate workplace in Sweden, Rosqvist (2019) identified a mode of engagement she termed “interest-based sociality” that occurred in autistic-only environments:

[I]interest-based sociality should here be seen as intrinsic group sociality, as a motivator and a driving force for social interaction within a group and a sense of belonging within a community. It includes the importance of having interest-based exchanges with one another, and having common interests and communication based on genuine interest in the topic being discussed. (Rosqvist, 2019, p. 176)

The exchange about his dog in the matched-dispositional condition seems to fit this description. A1 offers up a special interest that is of great importance to him and it is both recognized and reciprocated by A2 who is also passionate about her own dog. It would be tempting to assume some linear correlation between the engaging in a passage of autistically-satisfying interest-based sociality and the ensuing high affect and flow that characterize this conversation. However, the synchrony was already occurring before this episode: a degree of *tuning-in* already appeared to be taking place.

### Suite Two

#### Suite Two *Flow* and *Tuning in*

Suite Two continues with the presence of heavily monologic turns. The familiar matched-dispositional^6^ conversation between A3 and her chosen conversation partner X3 (an autistic friend made through Assert), for example, has an opening turn of 37 lines (lasting 1 min and 8 s), peppered only by X3's minimal backchanneling (“mmm, hm mm”). While A3 does tend to dominate the conversational flow (in all three of her conversations), X3 also inclines toward longer turns. At the end of A3's long opening sequence, having invited a response from X3 (“I don't know about you?”), X3 then goes on to hold the floor for a 60-line extended sequence (lasting 1 min 34 s) with just minimal backchanneling from A3.

“Monologues” are one of the examples given under the diagnostic criteria relating to a “failure of normal back and forth conversation” in the DSM-5 (American Psychiatric Association, 2013). While such one-sided verbosity may seem at odds with maintaining conversational flow, in this conversation at least, it does not appear to cause significant disruption. This may because, as McDonnell and Milton (2014, p. 44) have asserted, autistic people “will often feel more in their flow when engaged in monologs or serial monolog style conversations… a practice sometimes engaged in when people on the autism spectrum talk to one another.”

Despite the length of each speaker's sequences, the other remains engaged throughout with a sense of rapport, demonstrated by lots of backchannelling, and mutual, enthusiastic agreement. During a passage where X3 is describing how she has found the city much easier to navigate during moments when traffic has been stopped, there is a moment of mirroring of the word “kindness.”


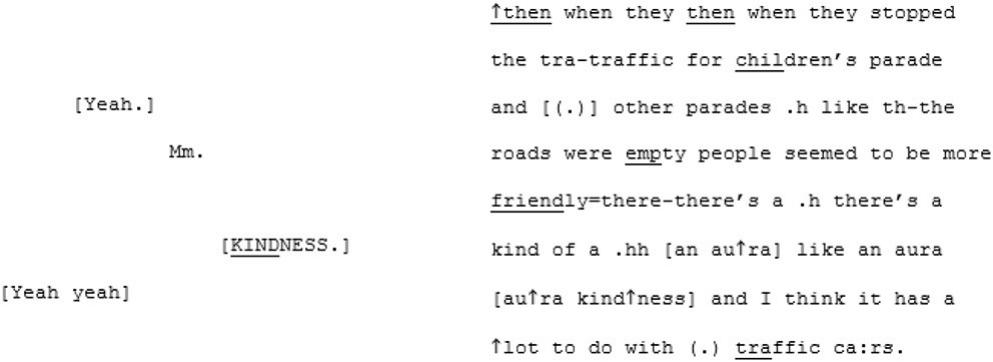


It could be the case that A3 has heard “a kind [of]” (line 56) and wrongly anticipated “kindness” as the coming word. However, while this was not the original word that X3 was working toward, it does seem that A3 has correctly understood the sentiment which is then mirrored back by X3.

The intersubjective synchrony that they appear to share, despite the (on first glance) stiltedness caused by the long turn-taking, is perhaps demonstrated most beautifully at the end of the conversation where they talk about the welcoming, sanctuary-like quality of the café that X3 frequents:


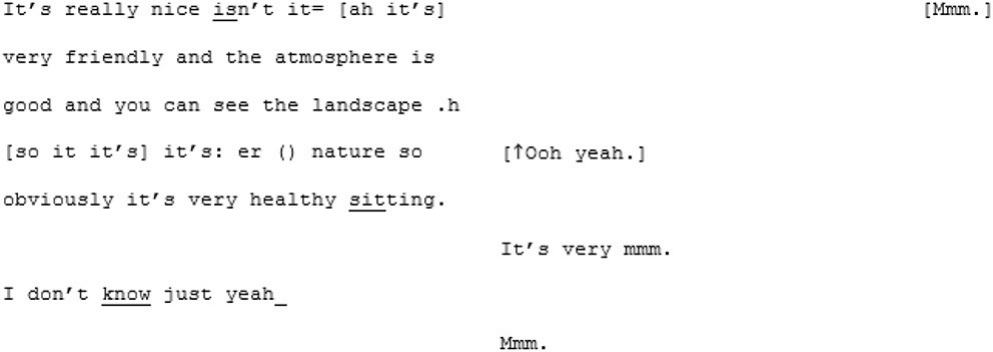


In lines 342–344 neither specifies what it is about that café that is of significance or value, or how this somehow functions as a supportive feature toward resilience against loneliness: but they both appear to “get” it. In this moment, whatever that quality of the café might be: it is mutually manifest to both A3 and X3. It is because of this that neither needs to spell it out.

These two speakers appear to be closely attuned. Their monologic turns do not disrupt the flow, perhaps because of the adjacency: both speakers are inclined to take them. The conversation has its own rhythm, its own flow and a sense of symmetry. Progressivity, here, is not rushed; each speaker allows the other to go on whilst maintaining the thread. There is a feeling of natural, structural coordination which may supports the building of “we-ness.”

The conversation between A4 and his non-autistic wife, X4 (familiar cross-dispositional condition), presents a very different conversational dynamic. This conversation, for the most part, involves fairly equal, short, and fluid turns. Yet despite this, attunement, rapport, and mutual understanding appear to be low throughout. Unique to this conversation is the proliferation of questions posed to check that they have been understood by, and have properly understood, the other (e.g., “Is that right? Is that what you're saying?”; “Are you talking about…”; “…does that make sense?”). This type of checking-in is often indicative of interlocutors who to wish signal investment in mutual understanding, and demonstrate care and attentiveness. However, combined with moments where A4's attempts at humor seem to fall flat, it might be interpreted as representing two individuals who are struggling to connect. Instead, our interpretation is that it is reflective of the fact that these interlocutors have a long personal history together and have perhaps learnt that in order to understand one another, extra effort must be made. They may know that they often don't understand each other at the first pass and are keen to monitor mutual understanding as conversation progresses.

These speakers, even in these short 10 min of dialogue, describe very different lifeworlds. A4 prefers to spend time by himself, hates parties and struggles to understand what loneliness would feel like. X4 takes pleasure from socializing, likes participating in organized groups and clubs and comes across as very in tune with her own feelings. While it may be the case that they have a lot of shared life experience together, their subjective experiences of the world—their dispositions—sound very different.

Their apparent difficulty in achieving mutual understanding is epitomized in the extract below where they struggle to understand what the other means, particularly around the definition of “loneliness.” A4 has repeatedly been saying that he doesn't “know what the word loneliness means” or what it “feels like”^7^. X4 seems to believe A4 just doesn't experience it as he doesn't need the company of others. From line 222 they fall into trying to define the concept of loneliness. X4 attempts to tell an anecdote describing a moment in which she felt lonely. A4 argues that what she is describing isn't “loneliness.” Suddenly the pace changes and where there was a balanced, measured exchange there are now rapid, over-lapping turns:


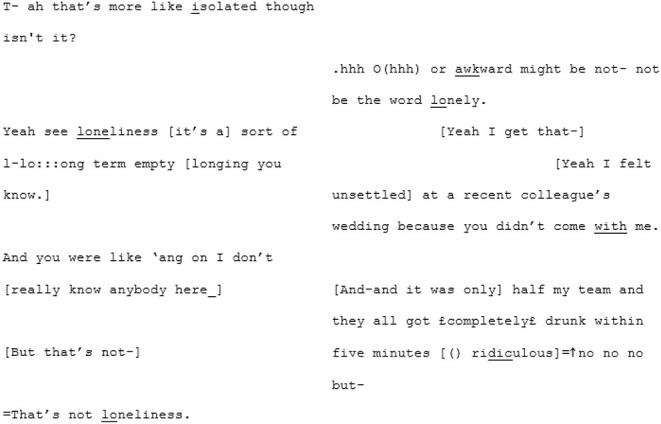


This sequence continues with A4 increasingly taking the floor until he interrupts X4 as she begins to respond and more or less continues in monolog form until the time is up, with very little further input from X4. The lack of understanding over what is quite a central issue to this conversation (loneliness), and this inability to synchronize leads not only to a breakdown of mutual understanding but a powerful breakdown of *flow*, and possibly, for this brief moment, rapport.

The unfamiliar matched-dispositional condition (where A3 and A4 meet) seems to have a very different quality. Here again, like in the familiar matched-dispositional interaction, two speakers with the potential for long turns are engaged in conversation, but it seems to *flow* effortlessly from the outset. There is a pace to this conversation, with over-lapping turns that seem to be borne of enthusiastic backchanneling and mirroring of what the other has said, often becoming direct echoing of words or phrases, as demonstrated in the following three short extracts:


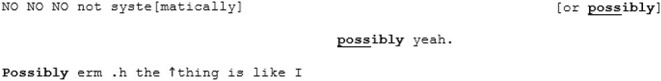


       ^**^


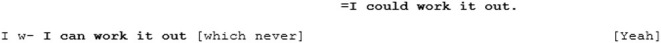


       ^**^


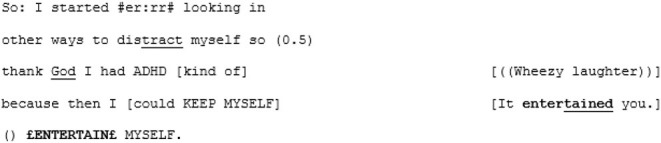


The most striking feature of this conversation, however, and most indicative that these speakers are *tuning-in*, is the immediate and enduring presence of humor and shared laughter, demonstrated in the final extract above. The humor appears to expands a sense of “solidarity” and rapport in which a deeply personal exchange was able to take place (both participants also shared how moving and surprising they had found the experience shortly after recording).

This use of humor to draw an interlocutor into synchrony contrasts with the way in which humor is used by A4 elsewhere. In the unfamiliar cross-dispositional condition (with B3), for example, A4's humor predicts and then undermines B3's earnest attempt to talk about her recent mental health difficulties and the reason she wanted to contribute to these conversations about loneliness:


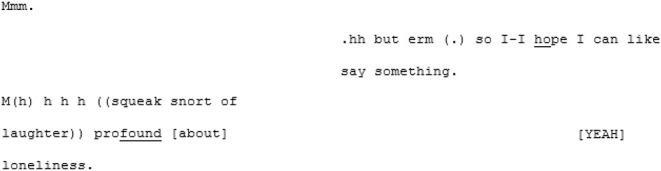


Following this deflective response, B3 ends her attempt to talk about the loneliness she had recently experienced and A4 takes the floor and, whether intentionally or not, this turn acts to maneuver the conversation away from potentially emotive content to a shallower sequence about loneliness facts and statistics.

Humor, then, seems to be utilized in different ways by A4 in these different conversational contexts to achieve different ends. In interaction with non-autistic stranger B3, he appears to be diverting the undesired direction of the conversation, moving it away from the potential intimacy with a stranger. In the familiar cross-dispositional condition (with X4) it comes across as (albeit affectionate) mocking. But it would not be fair to say that A4 consistently employs humor to avoid challenging emotional content, for in the unfamiliar matched-dispositional condition (with A3), humor rises up into natural exuberance, indicative of the spontaneous rapport and in the same conversation he leaves compassionate space for A3 to weep, and to share some of her childhood trauma:


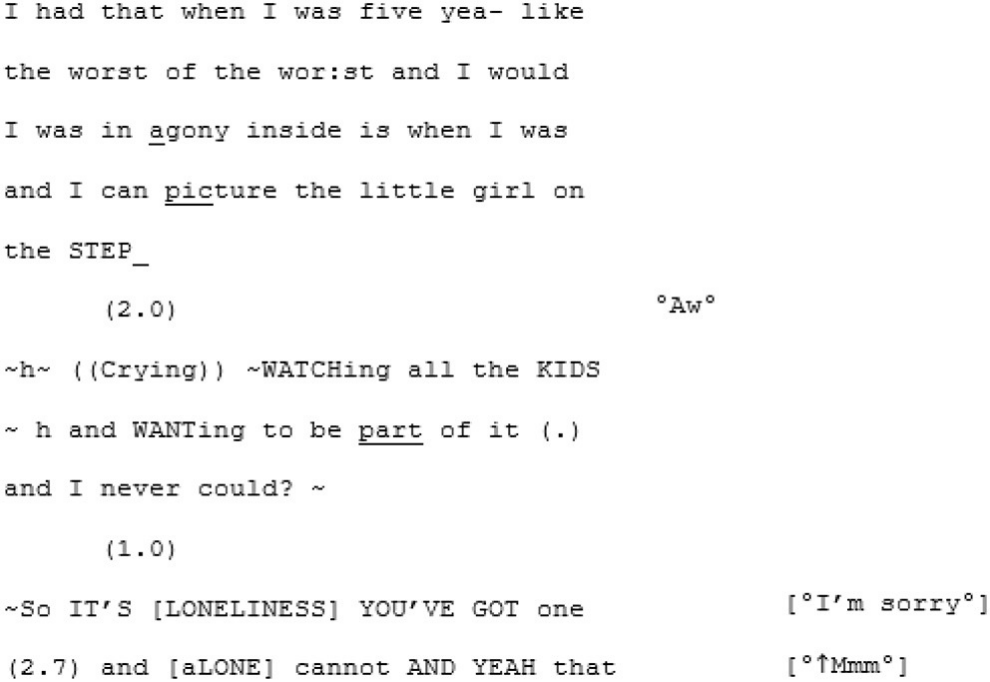


While A3 finishes her story over the following 10 lines, A4 quietly listens. There is no awkwardness, no attempt to interrupt or disrupt the flow with deflective humor and no stilted pauses when she has finished. Historically, this kind of muted response might have been interpreted as evidence of an autistic lack of interest in the feelings of others. Yet we suggest that this moment does not represent an absence of affective empathy. It is a moment of deep listening: of “daring to go on” (Sterponi and Fasulo, 2010) with A3 and her intimate sharing.

#### Suite Two *Running Along the Edges of Meaning*

Directly following the extract above, A3 completes her turn by explaining that her coping method, as a young child, was to turn to books. A4 responds by offering his own parallel anecdote, telling A3 how he also read a lot as a child, and used it as a way to access fantasy worlds: “faraway lands and magic and stuff that was all miles and miles away from what was a very isolated childhood I think” (lines 206–209). For someone who has repeatedly expressed uncertainty around the concept of loneliness and what it means for him, this seems an insightful moment. It spurs A3 to share a memory of a book that was special to her, which triggers a creative, playful, exuberant sequence:


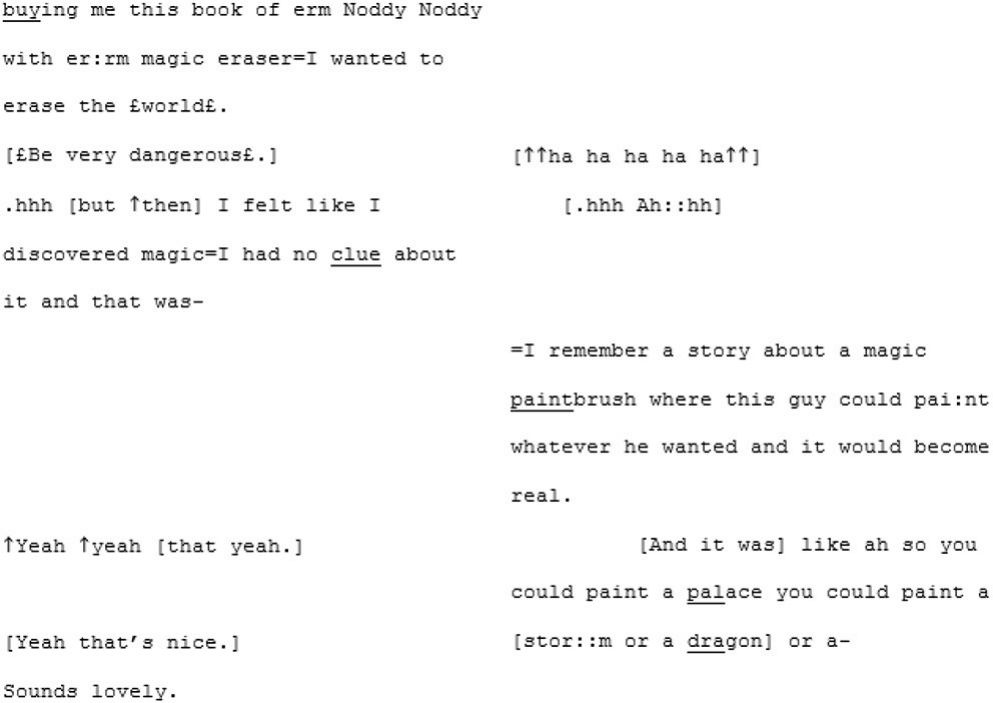


What makes this sequence so joyful, and powerful, is the fact that they have both dared to play. There is a feeling of an engagement of trust in the other's utterances, scaffolding progressivity out beyond the normal bounds of polite conversation into childlike creativity. They have entered what Sterponi and Fasulo (2010, p. 131) might refer to as a “liminal conversation space.” Here, the world—that may, at times, have been experienced as hostile and unwelcoming—can be changed with the flick of a paintbrush or the swish of an eraser.

### Suite Three

#### Suite Three *Flow* and *Tuning in*

Suite Three also features two core participants (A5 and A6) who demonstrate a tendency toward long turns. In the case of A6, her long turns seem to occur as a result of her laboring a little over formulating concise sentences. Like with A1's speech, there are false-starts, fillers, re-phrasings, multiple pauses occasional stutters in her conversation with X6 (familiar cross dispositional condition):


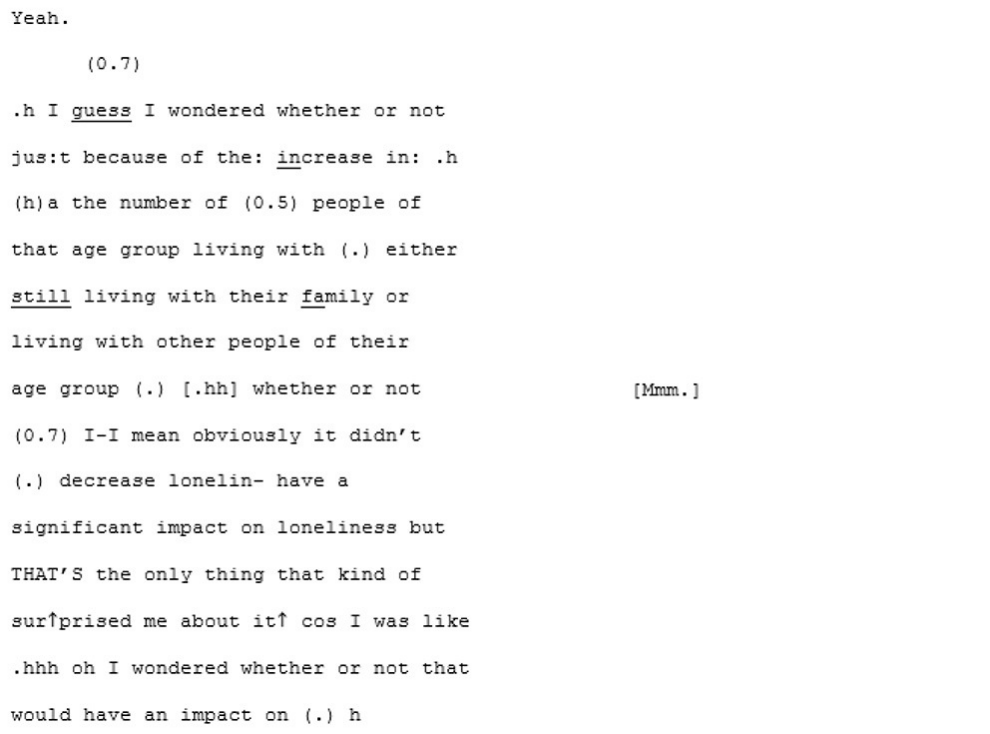



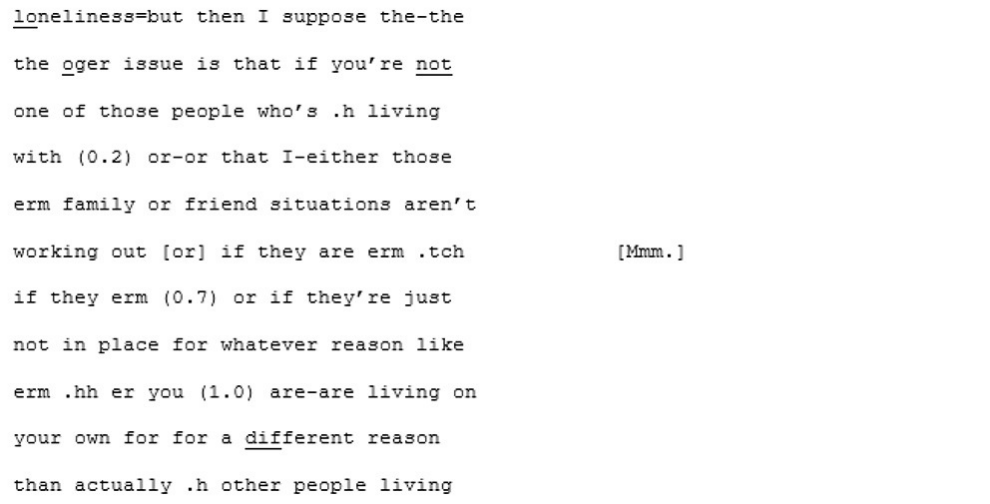



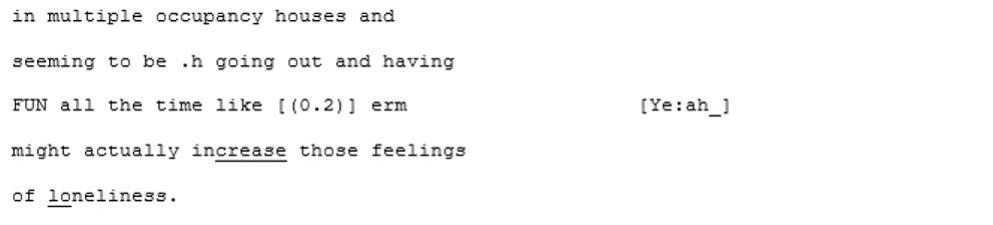


These features all combine to stall the flow of A6's speech and the pace of the exchange. Where A6 interjects with supportive or enthusiastic backchannelling when her friend (X6) is speaking, X6 tends to sit back when A6 is engaged in formulating a long, sometimes meandering turn. On first glance this may seem like disengagement, but this conversation also seems to feature some moments of affective coordination in the form of shared laughter and cooperative sequences where both parties' turns build toward a shared perspective.

The unfamiliar cross-dispositional conversation 15 (where A6 meets B4) provides a useful comparison. There are just a couple of moments fairly early into the conversation where B4 interjects while A6 is speaking. These interjections are phatic agreements, but because they are more substantial than X6's simple “Mmms” (in familiar cross-dispositional condition) they arguably require more processing effort.


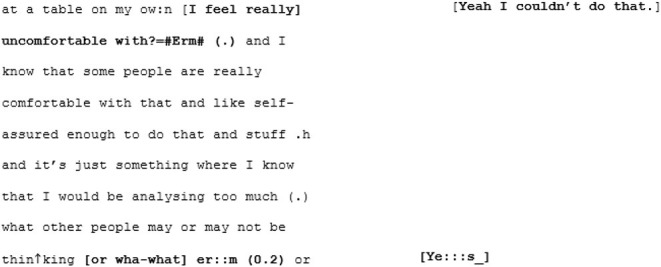


On each of these occasions (lines 112–113; line 120), the interjection appears to cause A6 a disruption in her train of thought, triggering a stutter, a filler, a pause. Although the interjection in line 120 (“yes”) is only a single word, it is delivered elongated and with flat intonation, marking it as somehow salient and requiring additional processing effort to derive the intended effects (such as an implied attitude or an intention to take the floor). These moments where one is required to simultaneously produce an utterance and process an incoming one can be hard for individuals with a monotropic disposition (i.e., with tightly focused, rather than diffuse, attention). Particularly for those individuals who also have sensory processing difficulties—where parsing speech among a competing cacophony of other (potentially informative) sounds is challenging—a cognitive lag may ensue at moments of high-speed task-switching. These temporary derailments do not seem to affect the potential for rapport. What these two conversations together (both cross-dispositional) perhaps demonstrate is that X6's subdued interjections may be reflective of her familiarity with her friend's need for space when constructing a complex utterance.

A6's second conversation (in the unfamiliar matched-dispositional condition, with A5) begins with a long turn, with no backchanneling from A6 whatsoever until line 26, and then only a handful of backchannels “Mmm”s or “Yeah”s for the remainder of A5's long turn (in total lasting 52 lines/1 minute and 44 seconds). Ordinarily this might indicate minimal engagement. In the context of A6 potentially requiring more time to process linguistic inputs (as discussed above), it might be tempting to wonder whether she is taking time to acclimatize to the language use of a novel interlocutor. Yet A6 begins her first turn (in line 53) by answering with a series of short responses, almost list-like, in response to the points A5 has made. It is here (lines 53–86) that the pace begins to pick up with A5 acknowledging each of A6's comments enthusiastically, creating what might be described as a conversational volley.

Perhaps it is the momentum that has been building that sets the stage for synchrony, but in the following sequence the pair arrive at a moment of mutual understanding—of mutual manifestness—around the meta-perspective-taking of an imagined other:


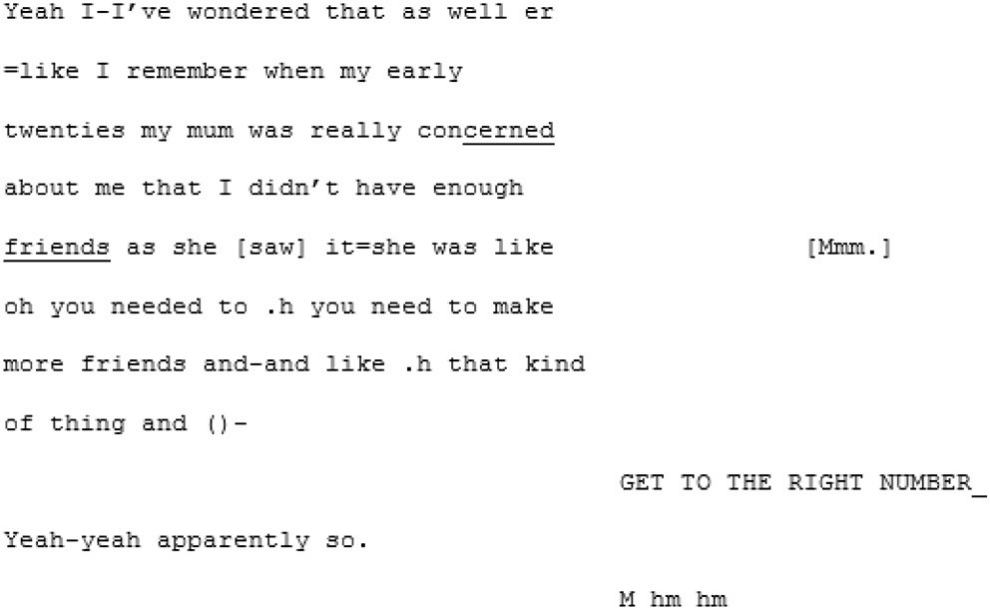


Here we assume that this hypothetical “other” (based, initially, on A5's mum) is non-autistic, and this is where the niche of this particular moment of mutual manifestness works. In this moment the othering routinely experienced by autistic people is flipped, and A6's “GET TO THE RIGHT NUMBER” is an echoic parodying of an imagined non-autistic perspective. A shared in-joke is created, based on the shared and unifying experience of being judged by an external “normative” perspective that both speakers can (a) speak to and (b) safely assume their interlocutor, being autistic, is also familiar with.

From here the conversation flows into a dense sequence of apparent close attunement with overlapping turns where they are not so much echoing each other as speaking in sync:


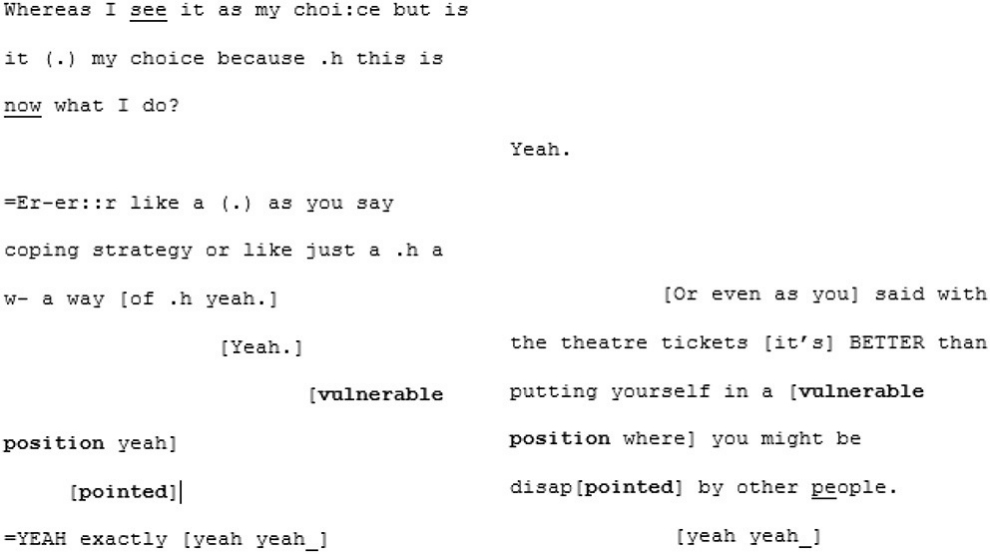


Most distinctive about this next phase of the conversation, however, is the dramatic shift in fluency of A6's speech. The stumbles, the re-starts and the drifting, long utterances are almost immediately eradicated and in their place, there is a concise, assured voice:


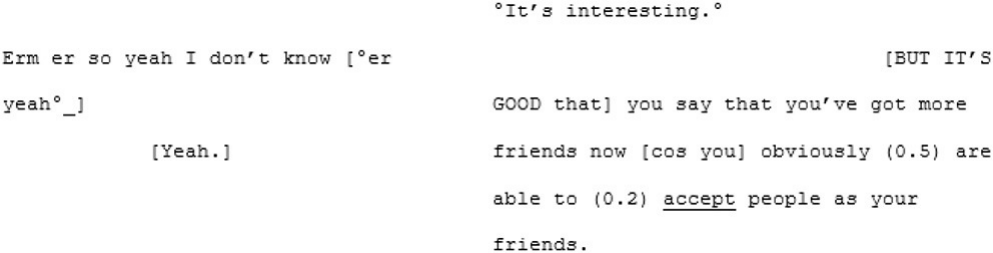


       ^**^


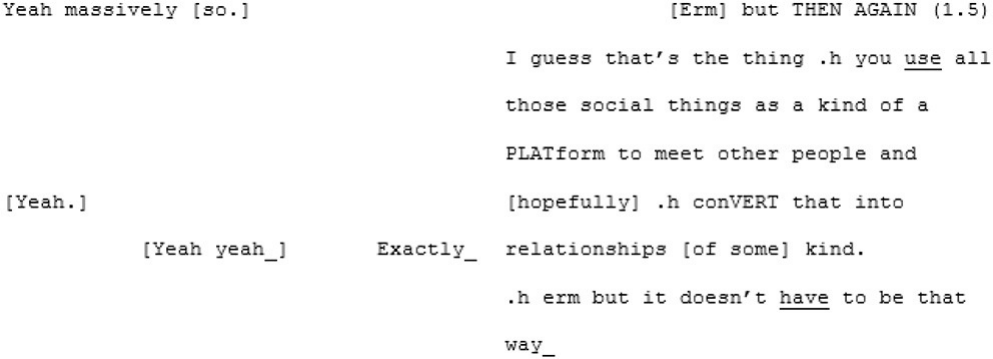


One possible explanation for this increase of flow of A6's own speech is that this is now her second conversation so she has had time to shake off any initial nerves associated with being recorded. However, as we saw above, in the subsequent unfamiliar cross-dispositional condition, she reverted to the earlier lack of fluency.

The rapport, flow, and attunement (in the form of backchanneling and agreement), remain until this conversation closes shortly after, as does A6's new-found ease of expression. This high level of rapport, attunement, and flow had not appeared to be present in A5's earlier conversation either. The familiar cross-dispositional conversation (between A5 and X5) seemed to lack flow, perhaps on account of the protracted monologic turns taken predominantly by A5. While some rapport was present (evidenced by moments of occasional phatic laughter, and consistent backchannelling throughout), it remained restrained.

In A5's final conversation, in the unfamiliar cross-dispositional condition, she meets non-autistic stranger B4. In contrast to the long turns with her familiar non-autistic conversation partner X5, it begins with a smooth sequence of shorter, interactive turns that flows easily, perhaps because she has come directly from the highly fluid matched-dispositional conversation with A6. Similar to the kind of subjective differences seen in the conversation between A4 and his wife X4, these two speakers describe very different lifeworlds. B4 likes “going out,” to the pub or to gigs and ideally in large groups. In contrast, she has had to work hard not to feel self-conscious being seen alone in public places (like a café). A5 tends to do things on her own. Yet this pair acknowledged and approached their differences with a kind of warm curiosity. They ask questions of each other: not “have you understood me?” but “tell me more…”:


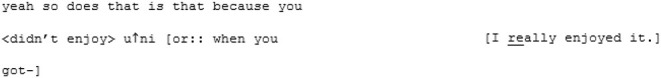


       ^**^


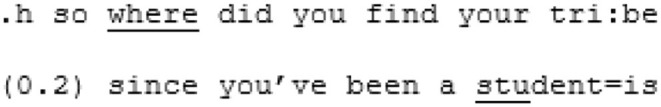


       ^**^


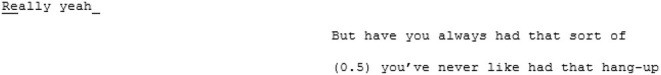


Very early on in the conversation, B4 shares the observation that her experience of being a student was quite lonely. As she put it, she had not been able to “find her tribe”^8^. In offering up this information, B4 exposes a degree of vulnerability from the outset. Considering these interlocutors are strangers, this is quite a bold move and one that invites intersubjective alignment and rapport. More than that—and not necessarily knowing that this might be the case—it sets the scene for common ground. While it is not expressed directly by A5 that she too experienced difficulty a community with whom she could connect, it is a common theme of autistic experiences.

By the time we reach adulthood, autistic people's experience of “togetherness” has likely consisted of some combination of: being intruded on by other people wanting us to engage with them, when we don't share that desire; being interested and curious about other people, but finding them confusing and overwhelming to be around; trying to engage with other people, and having frustrating and unsuccessful encounters; managing to engage “successfully” with other people, and finding ourselves drained and possibly even damaged as a result of what we had to do to “succeed.” (Sinclair, 2010, para. 3)

Here they appear to have inadvertently arrived at a means of bridging two mismatched dispositions: by naming, early on, a feeling of un-belonging that it is likely they both can recognize. Despite this conversation being both with an unfamiliar interlocutor and in the cross-dispositional condition, there is a far greater sense of *tuning-in*, as compared to the earlier conversation between A5 and X5 (familiar cross-dispositional). With its rapport, affect, and synchrony it seems to establish a sense of we-ness that might serve as a temporary community, with all the nourishment that that might bring.

### Suite Four

#### Suite Four *Flow* and *Tuning in*

This final suite begins with a conversation between A7 and her elder sister (familiar cross-dispositional). Unlike many of the other core autistic participants, A7 does not dominate the floor with long turns: if anything the conversation is guided by X7 as she poses the questions and ventures points to discuss. The conversation lacks much enthusiasm or “spark” and, listening to the recording, both participants speak in low, quite hushed tones with a consistently flat intonation. In addition to the frequent cross-talk there are regular gaps and lapses.

However, both interlocutors seem keen to engage with the other and progress the conversation. They each contribute and respond relevantly to each other's utterances. Yet despite this, the conversation appears to flow like two strangers trying to dance and repeatedly, apologetically, treading on each other's toes:


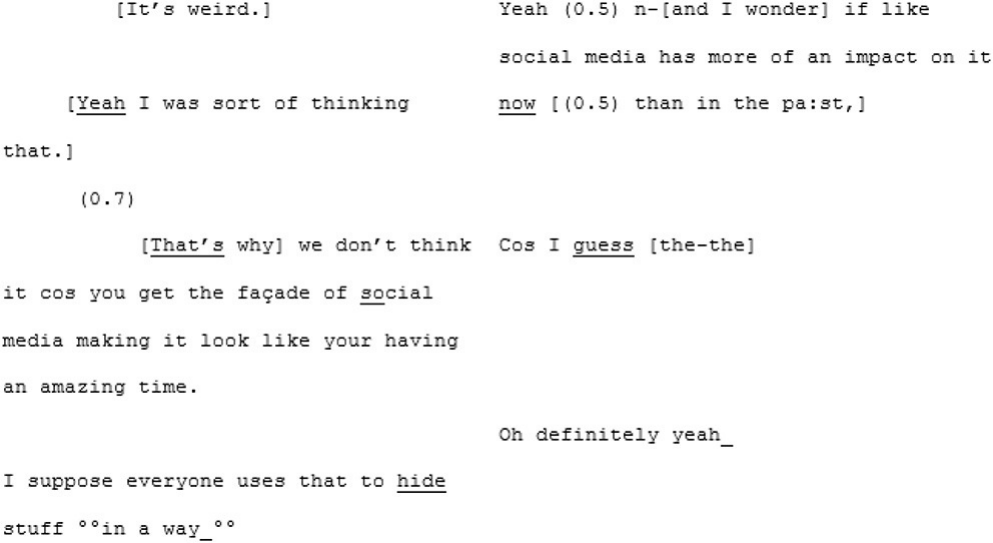


This lack of *flow* also seems to corresponds with an absence of *tuning-in*, perhaps because, as we saw in the cross-dispositional condition involving A4 and his familiar partner, these two speakers have quite different life experiences and lifeworlds despite being sisters. X7 has settled into married life and lives with her husband and two very young children. She, too, grew up in Brighton and now often bumps into old and new friends when she's walking around. A7 lives in a shared house, has very few friends and in spite of trying hard to meet people in organized social activities (“meet-ups”), finds it hard to make meaningful connections.

A7 has been explaining that she not only finds it hard to meet people she can connect with in Brighton, but that the fact she grew up locally makes her feel more self-conscious about not having many friends here (“I feel like the weird one for being, like, I've actually grown up here. I've lived here most of my life but I'm lacking people even though I'm in Brighton”). X7 is making attempts to console A7, telling her that this lack of connection A7 is describing is really due to chance (and perhaps attempting to imply that it is therefore not attributable to anything intrinsic to A7).


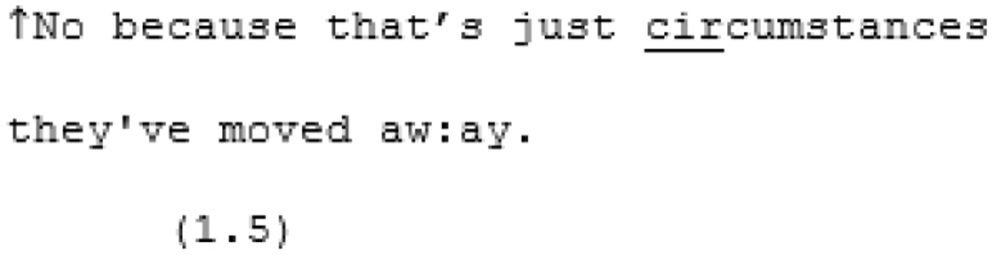


A7 seems to be trying to express a sense of isolation and alienation from the wider society that can be a common experience for autistic people. Inadvertently, in trying to comfort A7, X7 may in fact be undermining A7's attempt to share her pain. This moment of missed mutual understanding continues as each continue to talk from their own conflicting perspectives.


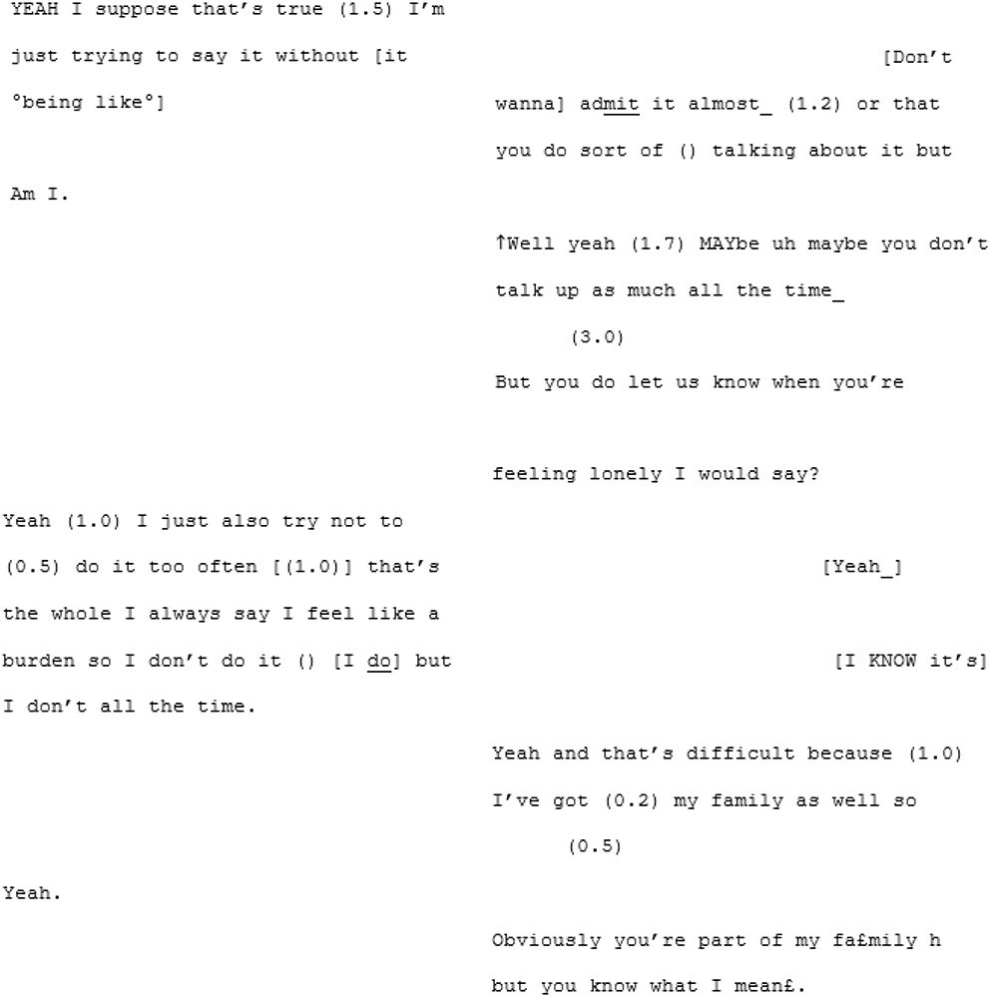


The lack of mutual understanding does not appear to stem from a lack of desire to connect. Here are two sisters who appear to care for each other a great deal but their dispositional difference, in this conversation, is seeming hard to bridge. While, in the above lines, A7 seems to be trying to voice a profound loneliness and a sense of not knowing how to reach out, X7 maintains the belief that A7 always lets them know when she is feeling lonely. What else can A7 really say other than “yeah…” (line 305). As the conversation draws to a close, and following X7's suggestion that A7 should send a text or even call someone if she felt really lonely, A7 tries one more time to make her sense of detachment from others around her understood:


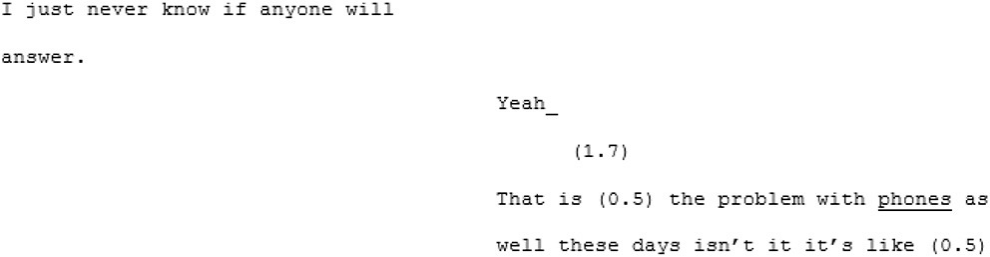


These speakers appear to be talking at cross-purposes. A7 is, seemingly, trying to talk about the unreliability of people while X7 is talking about the unreliability of modern technology. The information that A7 is working hard to convey is not mutually manifest here, leading to a breakdown in mutual understanding.

This all contrasts with the way in which the conversation involving A7 and B5 unfolds (the unfamiliar cross-dispositional condition). As with all the non-autistic stranger participants (“B”s), B5 does not know that A7 is autistic. Unlike the familiar cross-dispositional condition, where cross-talk was prevalent, here there is none. Turns are well-balanced, representing a consistently fluid back-and-forth. While A7 still has pauses mid-speech—where she appears to be preparing the next part of her utterance—there are very few of the lapses and gaps that punctuated the earlier conversation with her sister.

For A5 and B4 in the same conversational condition (unfamiliar cross-dispositional), the discovery that they had both experienced difficulty in finding a community they could belong to, opened up space for shared solidarity. In the same way, A7 and B5 also find several things in common, such as the invisibility to others of their deep loneliness and an aversion to socializing in a context fueled by recreational drugs (something they describe as common in the local social scenes). Around lines 78–89, B5 shares the observation that for her, one of the challenges of approaching new people is the fact that it's hard to know for sure whether they are a “good person” or not. Although A7 does not volunteer any further contribution to this topic, she does agree emphatically:


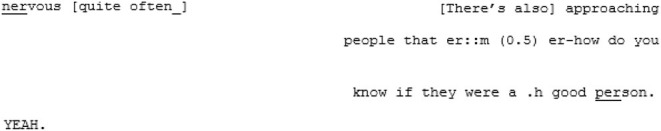


So-called “social naivety” has long been associated with autism (Lai and Baron-Cohen, 2015), and instances of interpersonal victimization (or “mate crime”) are unfortunately common among autistic people (Pearson et al., 2020). Whether or not A7 has had direct experience of this herself, she is likely to be at least aware of the potentially increased risks.

Finally, a further similarity between the unfamiliar cross-dispositional conversations of A5 and A7 respectively, is the way in which the opportunity for rapport and intersubjective alignment has been created by the sharing of some personal information by one of the speakers. B5, for example, talks about not having had a family growing up and how, now, it means that she doesn't “have people that I could just go to that just accept me and will listen to me” (lines 210–212). The sentiment expressed here sounds very similar to A7's “I just never know if anyone will answer” in her familiar cross-dispositional conversation [and, incidentally, echoes A1's “when you phone it (the mental health helpline) no one ever answers”]. It is the “daring to go on” (Sterponi and Fasulo, 2010) by making some private aspect of the self-visible, that invites the possibility for mutual understanding on a deep level.

#### Suite Four *Running Along the Edges of Meaning*

In the conversations involving A8 there is a distinct lack of *flow*, although the extent to which *flow* is disrupted varies between conversations. There is something idiosyncratic about A8's speech that sometimes can make it challenging to parse as a reader: but in the real-time back and forth of each conversation his interlocutors do not appear to notice directly. Structurally, A8's speech can jump at times between propositions that are not clearly coherent, but the difficulties occur most frequently at (and sometimes within) the level of a single word.

The precise nature of these errors is not clear from the speech sample available, and we had no access to any detailed assessment of speech and language, nor know whether this participant has ever had contact with speech and language therapy services. The errors may represent a developmental pattern of a speech sound disorder which A8 could have had since childhood. Speech sound disorders, while under-investigated in autistic people, have received increasing attention (see Wolk et al., 2016). Equally possible, however, are that these errors may be “paraphasias:” the term given to the presence of errors in an individual's speech, sometimes as the use of wrong words (“verbal paraphasia”), sometimes as wrong or switched phonemes (“phonemic paraphasia”) or sometimes as half-correct words (“neologistic paraphasia:” Millea, 2013). Although far less discussed than, say echolalia, paraphasia is also associated with autism, and there appear to be instances scattered among A8's speech (e.g., “everything” for anything, and “seeper” for cheaper, Conversation 18, lines 67 and 135; “meed” for mean, Conversation 20, line 68; and numerous verbal paraphasias)^9^.

Seen on the page these instances of word-level differences may jump out as odd or disruptive. Yet most of them are easily interpreted within the context of the surrounding utterance. At high-speed, given that a listener is already predicting what will be said before it has been spoken (Kikuchi et al., 2017), they may easily have gone unnoticed. It is possible, however, that they do contribute to the general stiltedness that colors these three conversations, not least because the occasional re-starts and re-phrases indicate that A8 is, to some extent, aware of these mis-speaks and attempting to monitor them. To do this, whilst also following his interlocutor's speech and crafting his own responses, is likely to add to the cognitive demand. It is little surprise that this might entail extra processing time in the form of pauses, gaps, and lapses.

A8's first conversation, in the familiar cross-dispositional condition with X8, lacks flow; there are a lot of gaps and lapses, frequent topic changes, and seemingly missed opportunities to extend or directly respond to what the other has said. Overall there is a sense of rhythmic awkwardness, as if both of them wish to keep the conversational ball in the air, but are finding it difficult to do so. For example, early in the conversation X8 shares an anecdote from when she had been walking recently in the countryside and was greeted by a stranger. A8 attempts a parallel response about how similar things happen when he goes for a walk near where his parents live, but stumbles a little and his response lasts just three lines (“Yeah cos with my parents are they… you they… you know if you…go on a walk… there…most people say hello”). There is a short lapse, then A8 re-takes the floor (“But going back to London…”). He proceeds to comment on something he has heard about London lacking racial integration, but it comes out awkwardly:

“People with the same backgrounds stay together so like, whites would stay together and Asians would stick together and all that. There's no, like, I could be wrong but there's no re-interaction between mixed races…”

The sometimes abrupt topic shifts between turns seen in this conversation give the impression of two parallel dialogues maintained over several turns. This dynamic is far more pronounced in the unfamiliar cross-dispositional condition where A8 meets non-autistic stranger B6. Unusually for these conversations, it is not the autistic participant (here, A8) taking long, monologic turns but B6. From the outset, B6 seems to dominate the conversational flow; his first turn is 50 lines long (lasting 2 min and 4 s), interjected only by one “Mmm” in line 35. This becomes a pattern during B6's long turns, where A8 provides minimal backchannel support but does not direct the conversation. It seems possible that A8 lets B6 run on because he is not entirely following B6's points. In his other conversations (familiar cross-dispositional and unfamiliar matched-dispositional), A8 tends to interject yet during B6's extended opening turn, A8 does not make use of many and ample pauses mid-flow. When he eventually re-enters the conversation (line 55), he initiates a new topic where he explains how long he has lived in Brighton and who he knows here, punctuated by several pauses. He then acknowledges B6's previous contribution (“…but it's a, it's an interesting point what you made, erm”), but picks out the incidental mention of the word “London” from much earlier in the conversation, rather than the B6's most recent point that he has experienced a lot of loneliness while being at university (“…but it's a, it's an interesting point what you made, erm, I mean the London, I don't go to London that often but I, they don't speak to each other on the tube they just listen to music”).

In the moments throughout the rest of the conversation with B6, when A8 does step in and take the floor, it appears to be to re-orientate the discussion back to a question related to the prompt cards (e.g., thinking about potential solutions to loneliness locally). In the same way that, in the familiar cross-dispositional condition, A8 and X8 would acknowledge each other's contributions but attempt to pursue a new direction, this conversation only just hangs together in terms of coherence.

In the unfamiliar matched-dispositional condition, where A7 and A8 come together, the conversation seems to have a more stable central point of gravity than A8's other two conversations. There is a symmetry in turn-taking and progressivity of the conversation and despite the still-present gaps, pauses, and lapses on the part of both speakers, this conversation nevertheless seems to *flow*. The conversation begins with the pair cooperating, *via* a series of short turns, to establish a joint definition of “loneliness:”


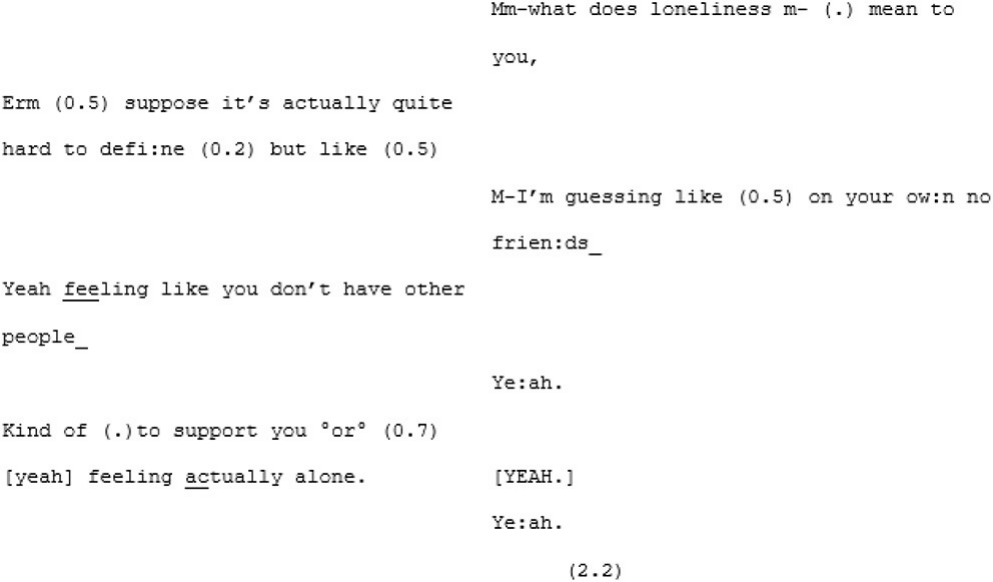


A8 poses some questions for A7 (“have you ever experienced loneliness in Brighton and Hove at all;” “do you know people or can you talk to people here?”) that, although they are perhaps a little stilted and led by the prompts, remain relevant and cohesive with the previous turns. A little later, A8 shifts topic again, asking A7 whether she thinks things like meet-ups might help to address loneliness in Brighton and Hove (a topic that he attempts to raise again in the subsequent conversation with B6, to no avail). Fortuitously, A7 has some experiences with meet-ups, as she described in the earlier (familiar cross-dispositional) conversation with X7. This triggers a fluid exchange that continues across 101 lines and 17 turns (and lasting 2 min and 28 s) divided across both speakers. This passage evolves naturally from meet-ups, to the time and money required to do them, to the working hours they both have, to how work in various sectors impacts on the ability to socialize. It is perhaps significant that the discovering of a shared interest initiated this extended, fluid passage of interaction.

What marks this conversation out from the other two in which A8 participates, is the fact that he appears able to sustain focus and coherence for far longer stretches. Moreover, his contributions are more directly relevant. While the enthusiastic rapport that we have seen in some of the other pairings seems to be lacking here, so too is the sense of awkwardness that is sometimes present in both the cross-dispositional conditions (familiar, with X8 and unfamiliar with B6). It is difficult to assess exactly what it is that makes this matched-dispositional conversation with an unfamiliar person function more successfully. There could simply be some degree of luck in A8 introducing a topic (meet-ups) that has some resonance with A7. Given that the other topic-related sequences also run on though, there is probably something else occurring here too. In their study investigating neurodivergent intersubjectivity, Heasman and Gillespie (2019, p. 910) found that conversations involving only autistic interlocutors had “a low demand for coordination that ameliorated many challenges associated with disruptive turns.” It may be that in this matched-dispositional condition there is implicitly less pressure for A8 to provide highly contingent contributions at all times and that this, ironically, allows him the space to provide them.

## Discussion

This study sought to investigate how implicit expectations of shared relevance contribute to breakdowns in understanding between autistic and non-autistic interlocutors. Eight core autistic participants engaged in three short conversations about loneliness: with a chosen, familiar conversation partner (“X”), with an autistic stranger (“A”) and with a non-autistic stranger (“B”). Mutual understanding was unexpectedly abundant during these conversations across all types of conversation pairings.

Clear patterns emerge when the four Suites of conversations are considered together. The most striking of these is the difference between conversations that involved two autistic participants (i.e., the matched-dispositional conversations) and those that involved cross-dispositional pairs. All five matched-dispositional conversations seem to be characterized by a significant (and sometimes dramatic) increase in flow, rapport, and intersubjective attunement. Conversations 3, 6, and 8 are colored brightly by enthusiasm and mutual affect. In contrast, all but a few of the conversations with non-autistic participants lack the above, even when interlocutors were well-known—and had been for a long time—to the core autistic participant.

The fact that interlocutors built rapport, flow, and synchrony far more effectively when both parties were autistic, even when they were strangers, seems to support theories that suggest we get on best with people who have similar minds (De Jaegher, 2013; Bolis et al., 2017; Fein, 2018; Chapman, 2019; Conway et al., 2019a,b). This, in turn, adds to evidence that counters the ToM-deficit theory of autism and bears out anecdotal evidence from autistic people that they sometimes find barriers to social communication minimized when engaging with other autistic people. For example, autistic academic Sinclair (2010, para. 42) observes that “the ‘same planet' metaphor, along with metaphors about ‘speaking the same language' or “belonging to the same tribe” are very common descriptions used by autistic people” who have had the opportunity to experience an autistic-dominant space. Similarly, autistic participants reported finding matched-dispositional interaction (i.e., with other autistic people) much more comfortable, in a study by Crompton et al. (2019a). Finally, while it perhaps shouldn't need to be said, the very presence of the high rapport and mutual interest demonstrated in these conversations contributes to the literature that challenges the reduced social motivation hypothesis of autism (Chevallier et al., 2012).

One further pattern is that some autistic participants (A1, A6, and A8) appeared to experience optimal individual communicative competence when engaged in exclusively autistic dyadic conversations. For example, A1's turns are shorter and similarly more coherent in the matched-dispositional conversation compared to the cross-dispositional interactions, contributing to a fluid progression of adjacent turns as opposed to the parallel dialogue of his previous conversation in his familiar cross-dispositional conversation condition. Similarly, when talking with A5, A6 is dramatically more fluent. Stumbles, pauses and re-starts that characterize the typically long utterances of the other two conversations are almost entirely absent and replaced with concise, cogent turns. Conversation 19 is the only one of three where A8 was able to maintain prolonged sequences of engaged, coherent turns.

This finding potentially lends support to a monotropic theory of autistic cognitive processing, explained by relevance theory. In those circumstances where increased mutual manifestness makes understanding less effortful (in both a technical relevance theoretic, and an intuitive sense), more cognitive resources are available for language production. Furthermore, according to the theory of monotropism, the attention of monotropic individuals is not simply narrowed, but also sharpened (Murray et al., 2005; Murray, 2018, 2020). In states of “monotropic superdrive” (Murray et al., 2005, p. 143) finer-grain details may carry heightened relevance. It seems possible that when two monotropic individuals synchronize their “torch-beams” (Murray et al., 2005, p. 140) of intensified attention, something like a hyper-confluence of cognitive environments may occur, with increased affective reward. This may explain, for example, why in a study involving an information transfer task (Crompton et al., 2019b), autistic people both transmitted the necessary information more efficiently and experienced higher rapport when interacting with other autistic people. These findings have potential implications for how the communicative competence of autistic people is assessed, particularly if assessing interlocutors are non-autistic.

Less common, but equally as important, are the moments where the gap between sometimes very different dispositions are bridged. The familiar cross-dispositional conversations involving A1, A6, and A8, while low on flow and at times asymmetrical, demonstrate how the familiarity of an interlocutor (X1, X6, and X8, respectively) can be functionally supportive where the autistic speaker struggles. In these conversations additional processing time was given, interruptions minimized and mis-speaks accommodated for. Yet it was during the conversations with non-autistic strangers where some of the most surprising moments of connection and mutual understanding were made. A6 and A7, with their respective unfamiliar cross-dispositional interlocutors managed to reach a state of attunement, flow, and rapport through the establishing of affective common ground. In the first instance this was achieved through warm curiosity manifesting in frequent questioning about the other's experiences, and in the second through the volunteering of personal information and emotional openness.

One potential reason for the high levels of mutual understanding across all conversations may be because speakers were orientated around a central topic (loneliness) which, having agreed to participate, they had an intrinsic motivation to address. If this is the case, it is not necessarily a limitation of this study: it points to the importance of creating engaging opportunities for interaction that match an autistic person's interests in order to support communication, something that mirrors findings by Koegel et al. (2013) and Wood (2019). This is further supported by the moments in these conversations where the discovery of a shared intense by pairs of autistic interlocutors sparked significantly increased conversational flow and interpersonal attunement. Another potential reason to consider is that participants may have become more accustomed to the task across the three conversation conditions. However, for six of the eight core autistic participants (all except for A7 and A8), the matched-dispositional conversation conditions where increased flow and attunement were observed came second, not third.

### Limitations and Directions for Future Research

As is often the case with rich, qualitative data, our sample size is small and would bear replication. In terms of method, the absence of a non-autistic-to-non-autistic pairing condition for the conversations may seem to be a short-coming, particularly for readers more accustomed to experimental designs. This, however, was a methodological choice. In their study analyzing patterns of intersubjectivity among small groups of autistic speakers, Heasman and Gillespie (2019, p. 910) chose to focus solely on the autistic-only interaction, arguing the following:

Autistic people are neurologically divergent, yet methods for investigating autistic sociality tend to assume neurotypical definitions of being social. Comparative design often results in autistic behavior being interpreted as a deficit, rather than a difference, from neurotypical benchmarks (Heasman and Gillespie, 2019, p. 910).

The aim of this present study was to investigate the strength of the hypothesis that the relevance theoretic notion of mutual manifestness might serve to support the double empathy problem theory of mutual misunderstanding in cross-dispositional communication. As such, our interests centered around analyzing the way in interactions unfolded in conversation taking place between autistic and non-autistic speakers. Some form of comparison was, of course, necessary and we felt that given our interest in the role of mutual manifestness, *familiarity* served as the most meaningful condition criteria (hence core autistic speakers were asked to bring a familiar conversation partner for their first conversation, and then were paired with an autistic stranger and a non-autistic stranger). However, in future replications of this study it may be interesting to include further conversation conditions, involving pairs of familiar and unfamiliar non-autistic speakers.

Perhaps the most important limitation of this study, however, relates to the sampling of participants: of whom all were white Caucasian. This occurred organically through the self-selection of the participants, though likely also reflects both the demographic of the city within which the research took place, and the diagnostic biases against autistic people of color and minority ethnicities (Begeer et al., 2009; Fein and Rios, 2018; Jones and Mandell, 2020; Cascio et al., 2021). This matters and not only because of the urgent imperative to shift the focus of autism research away from both the Global North and white-centric stereotypes. These conversations featured a high degree of rapport, conversational flow, and mutual understanding, but this all occurred within a white, mono-cultural context. Cascio et al. (2021) have noted the “double minority status” that some autistic people of color may experience: something that may further trouble opportunities for mutual understanding by reducing what is held in common. Further studies investigating intersubjectivity or the DEP may wish to address this, and actively include autistic people of color within the cohort. Additional implications for further research include replicating this study with a larger and more diverse cohort of autistic participants, as well as exploring the longer-term impact of therapies or interventions based around shared flow states on the pragmatic and prosocial abilities of autistic individuals.

Finally, there is an important caveat to be made in relation to the present study. Findings such as these, which indicate that autistic people may enjoy more synchronous communication with fellow autistic individuals, must absolutely not be interpreted as support for the exclusion of autistic people from “mainstream” society. Furthermore, findings from this study have not suggested that cross-dispositional attunement is an impossibility: quite the opposite. We hope that these findings might contribute to efforts to support and facilitate mutually satisfying cross-dispositional interactions.

## Data Availability Statement

The datasets presented in this article are not readily available because the ethical approval given for this study by the Tier II Arts and Humanities Ethics Panel at the University of Brighton covered the publication of anonymized extracts only. This was based on the understanding that even when identifying features are redacted from transcripts, conversation in full are inherently recognizable. As such, regretfully we are unable to provide transcripts in full. Requests to access the datasets should be directed to Gemma L. Williams, glwilliamsresearch@gmail.com.

## Ethics Statement

The studies involving human participants were reviewed and approved by Tier II Arts and Humanities Ethics Panel at the University of Brighton. The patients/participants provided their written informed consent to participate in this study. Written informed consent was obtained from the individual(s) for the publication of any potentially identifiable images or data included in this article.

## Author Contributions

CRediT contributor roles: GW, TW, and CJ: conceptualization, methodology, validation, writing—review, and editing. GW: data curation, project administration, and writing—original draft. GW and TW: formal analysis and investigation. GW: funding acquisition. TW and CJ: supervision. All authors contributed to the article and approved the submitted version.

## Conflict of Interest

The authors declare that the research was conducted in the absence of any commercial or financial relationships that could be construed as a potential conflict of interest.

## Footnotes

^1^In accordance with the preferences expressed by autistic self-advocates and their allies (see Kenny et al., 2016; Botha et al., 2021), this paper uses “identity-first language” (i.e., *autistic woman*) rather than person-first language (i.e., *a woman with autism*). This choice does not imply a negative judgement toward individuals with autism referring to themselves as such, if this is their wish.

^2^That our minds must be economical with what we notice in this vastly information-rich world is now fairly uncontentious. See, e.g., Gigerenzer and Todd (1999) or Clark (2013).

^3^See Leinonen and Ryder (2008) for detailed review.

^4^A secondary thematic analysis addressing the qualitative loneliness content—beyond the scope of this primary study—was undertaken and reported on in to ensure that this endeavor was indeed meaningful.

^5^N.B. There is certainly no intention to compare autism with schizophrenia, but in terms of communication there are potential parallels in the absence or reduction of mutual manifestness and the consequences that faulty assumptions around this, on the part of both interlocutors, may have.

^6^This conversational condition was unique to A3 as her chosen interlocutor happened to be autistic, unlike the chosen partners of the other core participants.

^7^This kind of response would be typical of an individual with alexithymia: a condition relating to the “difficulty identifying and talking about your own feelings” (Happé and Frith, 2020, p. 10) that frequently co-occurs with autism. The presence of such a condition, particularly if unidentified, would likely contribute considerably to difficulties in mutual understanding.

^8^We recognize that this term can be problematic, and potentially culturally appropriative but we wished to remain true to the participant's words and the spirit with which they were uttered.

^9^Here the word “wrong” does not connote any negative judgement.

## References

[B1] American Psychiatric Association (2013). Diagnostic and Statistical Manual of Mental Disorders: DSM-5, 5th Edn. Washington, DC: American Psychiatric Association.

[B2] Baron-Cohen S. Leslie A. M. Frith U. (1985). Does the autistic child have a “theory of mind”? Cognition 21, 37–46. 10.1016/0010-0277(85)90022-8 2934210

[B3] Beardon L. (2017). Autism and Asperger Syndrome in Adults. London: Sheldon Press.

[B4] Begeer S. El Bouk S. Boussaid W. Terwogt M. M. Koot H. M. (2009). Underdiagnosis and referral bias of autism in ethnic minorities. J. Autism Dev. Disord. 39, 142–148. 10.1007/s10803-008-0611-5 18600440

[B5] Bogdashina O. (2005). Communication Issues in Autism and Asperger Syndrome: Do We Speak the Same Language? London: Philadelphia, PA: Jessica Kingsley Publishers.

[B6] Bogdashina O. (2010). Autism and the Edges of the Known World: Sensitivities, Language and Constructed Reality. London: JKP.

[B7] Bolis D. Balsters J. Wenderoth N. Becchio C. Schilbach L. (2017). Beyond autism: introducing the dialectical misattunement hypothesis and a bayesian account of intersubjectivity. Psychopathology 50, 355–372. 10.1159/000484353 29232684

[B8] Botha M. Williams G. L. Hanlon J. (2021). Does language matter? Identity-first versus person-first language use in autism research: a response to vivanti. J. Autism Dev. Disord. 10.1007/s10803-020-04858-w. [Epub ahead of print]. PMC7817071 33474662

[B9] Braun V. Clarke V. (2006). Using thematic analysis in psychology. Qual. Res. Psychol. 3, 77–101. 10.1191/1478088706qp063oa

[B10] Braun V. Clarke V. (2020). One size fits all? What counts as quality practice in (reflexive) thematic analysis? Qualitative Res. Psychol. 10.1080/14780887.2020.1769238. [Epub ahead of print].

[B11] Brewer R. Biotti F. Catmur C. Press C. Happé F. Cook R.. (2016). Can neurotypical individuals read autistic facial expressions? Atypical production of emotional facial expressions in autism spectrum disorders. Autism Res. 9, 262–271. 10.1002/aur.1508 26053037 PMC4975602

[B12] Cascio M. A. Weiss J. A. Racine E. (2021). Making autism research inclusive by attending to intersectionality: a review of the research ethics literature. Rev. J. Autism Dev. Disord. 8, 22–36. 10.1007/s40489-020-00204-z

[B13] Chapman R. (2019). Autism as a form of life: wittgenstein and the psychological coherence of autism. Metaphilosophy 50, 421–440. 10.1111/meta.12366

[B14] Chapman R. (2020). The reality of autism: on the metaphysics of disorder and diversity. Philos. Psychol. 33, 799–819. 10.1080/09515089.2020.1751103

[B15] Chevallier C. Kohls G. Troiani V. Brodkin E. S. Schultz R. T. (2012). The social motivation theory of autism. Trends Cogn. Sci. 16, 231–239. 10.1016/j.tics.2012.02.007 22425667 PMC3329932

[B16] Chown N. (2012). A treatise on language methods and language-games in autism (doctoral dissertation). Sheffield Hallam University. Available online at at: http://shura.shu.ac.uk/7164/

[B17] Chown N. Robinson J. Beardon L. Downing J. Hughes E. Leatherland J.. (2017). Improving research about us, with us: a draft framework for inclusive autism research. Disabil. Soc. 32, 720–734. 10.1080/09687599.2017.1320273

[B18] Clark A. (2013). Whatever next? Predictive brains, situated agents, and the future of cognitive science. Behav. Brain Sci. 36, 181–204. 10.1017/S0140525X12000477 23663408

[B19] Conway J. R. Catmur C. Bird G. (2019a). Understanding individual differences in theory of mind via representation of minds, not mental states. Psychonomic Bull. Rev. 26, 798–812. 10.3758/s13423-018-1559-x 30652239 PMC6557866

[B20] Conway J. R. Coll M. P. Cuve H. C. Koletsi S. Bronitt N. Catmur C.. (2019b). Understanding how minds vary relates to skill in inferring mental states, personality, and intelligence. J. Exp. Psychol. Gen. 149, 1032–1047. 10.1037/xge0000704 31670565

[B21] Crompton C. J. Fletcher-Watson S. Ropar D. (2019a). “I never realised everybody felt as happy as I do when I am around autistic people”: a thematic analysis of autistic adults' relationships with autistic and neurotypical friends and family. Autism 24, 1438–1448. 10.1177/1362361320908976 32148068 PMC7376620

[B22] Crompton C. J. Fletcher-Watson S. Ropar D. (2019b). Autistic peer to peer information transfer is highly effective. Autism 24, 1704–1712. 10.1177/1362361320919286 32431157 PMC7545656

[B23] Cusack J. Sterry R. (2016). Your Questions: Shaping Future Autism Research. London: Autistica. Available online at: http://www.jla.nihr.ac.uk/priority-setting-partnerships/autism/downloads/Autism-PSP-final-report.pdf

[B24] Cushing S. (2013). “Autism: the very idea,” in The Philosophy of Autism, eds J. L. Anderson and Simon Cushing (Plymouth: Rowman & Littlefield), 17–45.

[B25] De Jaegher H. (2013). Embodiment and sense-making in autism. Front. Integr. Neurosci. 7:15. 10.3389/fnint.2013.00015 23532205 PMC3607806

[B26] De Jaegher H. (2020). Seeing and inviting participation in autistic interactions. OSF Preprints. 10.31219/osf.io/bhjfc 34591703

[B27] Di Paolo E. A. Cuffari E. C. De Jaegher H. (2018). Linguistic Bodies: The Continuity Between Life and Language. Cambridge, MA: MIT Press. 10.7551/mitpress/11244.001.0001

[B28] Edey R. Cook J. Brewer R. Johnson M. H. Bird G. Press C. (2016). Interaction takes two: typical adults exhibit mind-blindness towards those with autism spectrum disorder. J. Abnorm. Psychol. 125, 879–908. 10.1037/abn0000199 27583766

[B29] Elson P. R. Wamucii P. Hall P. V. (2018). Scaling up community-based research: a case study. Res. All 2, 374–392. 10.18546/RFA.02.2.14

[B30] Fein E. (2018). “Autism as a mode of engagement,” in Autism in Translation: An Intercultural Conversation on Autism Spectrum Conditions, eds E. Fein and C. Rios (Cham: Palgrave Macmillan), 129–154. 10.1007/978-3-319-93293-4_6

[B31] Fein E. Rios C. (2018). Autism in Translation: An Intercultural Conversation on Autism Spectrum Conditions. Cham: Palgrave Macmillan. 10.1007/978-3-319-93293-4

[B32] Fletcher-Watson S. Adams J. Brook K. Charman T. Crane L. Cusack J.. (2019). Making the future together: shaping autism research through meaningful participation. Autism 23, 943–953. 10.1177/1362361318786721 30095277 PMC6512245

[B33] Fletcher-Watson S. Happé F. (2019). Autism: A New Introduction to Psychological Theory and Current Debate. London; New York, NY: Routledge.

[B34] Gigerenzer G. Todd P. (1999). “Fast and frugal heuristics: the adaptive toolbox,” in Simple Heuristics That Make us Smart, eds G. Gigerenzer, P. Todd, and the ABC Research Group (Oxford: OUP), 3–34.

[B35] Grice P. (1975). “Logic and conversation,” in Syntax and Semantics 3: Speech Acts, eds P. Cole, et al., 41–58. 10.1163/9789004368811_003

[B36] Happé F. Frith U. (2020). Annual research review: looking back to look forward–changes in the concept of autism and implications for future research. J. Child Psychol. Psychiatry 61, 218–232. 10.1111/jcpp.13176 31994188

[B37] Happé F. G. (1991). “The autobiographical writings of three Asperger syndrome adults: problems of interpretation and implications for theory,” in Autism and Asperger Syndrome, ed U. Frith (Cambridge: CUP), 207–242. 10.1017/CBO9780511526770.007

[B38] Happé F. G. (1993). Communicative competence and theory of mind in autism: a test of relevance theory. Cognition 48, 101–119. 10.1016/0010-0277(93)90026-R 8243028

[B39] Happé F. G. (1995). Understanding minds and metaphors: insights from the study of figurative language in autism. Metaphor Symbol 10, 275–295. 10.1207/s15327868ms1004_3

[B40] Heasman B. Gillespie A. (2017). Perspective-taking is two-sided: misunderstandings between people with Asperger's syndrome and their family members. Autism 22, 740–750. 10.1177/1362361317708287 28683569 PMC6055325

[B41] Heasman B. Gillespie A. (2019). Neurodivergent intersubjectivity: distinctive features of how autistic people create shared understanding. Autism 23, 910–992. 10.1177/1362361318785172 30073872 PMC6512057

[B42] Hedley D. Uljarević M. Wilmot M. Richdale A. Dissanayake C. (2018). Understanding depression and thoughts of self-harm in autism: a potential mechanism involving loneliness. Res. Autism Spectr. Disord. 46, 1–7. 10.1016/j.rasd.2017.11.003

[B43] Holt-Lunstad J. Smith T. B. Layton J. B. (2010). Social relationships and mortality risk: a meta-analytic review. PLoS Med. 7:e1000316. 10.1371/journal.pmed.1000316 20668659 PMC2910600

[B44] Hubbard D. J. Faso D. J. Assmann P. F. Sasson N. J. (2017). Production and perception of emotional prosody by adults with autism spectrum disorder. Autism Res. 10, 1991–2001. 10.1002/aur.1847 28815940 PMC6061943

[B45] Interagency Autism Coordinating Committee (IACC) (2013). Autism Spectrum Disorder Publications Analysis: the Global Landscape of Autism Research. Washington, DC: Interagency Autism Coordinating Committee; US Department of Health and Human Services. Available online at: http://iacc.hhs.gov/strategic-plan/2013/index.shtml

[B46] Jagoe C. (2012). “Schizophrenia and metarepresentational abilities in conversation: a preliminary analysis of question interpretation from a Relevance Theoretic perspective,” in Relevance Theory: More than Understanding, eds E. Walaszewska and A. Piskorska (Cambridge: Cambridge Scholars Publishing), 261–278.

[B47] Jagoe C. (2015). Collaborative meaning-making in delusional talk as a search for mutual manifestness: a relevance theory approach. J. Interactional Res. Commun. Disord. 6, 53–70. 10.1558/jircd.v6i1.53

[B48] Jagoe C. Smith M. (2016). “Balancing multimodality and relevance,” in The Silent Partner? Language Learning and Language Use in Aided Communication, eds M. Smith and J. Murray (London: J&R Press), 229–246.

[B49] Jagoe C. Wharton T. (2021). Meaning non-verbally: the neglected corners of the bi-dimensional continuum communication in people with aphasia. J. Pragmat. 178, 21–30. 10.1016/j.pragma.2021.02.027

[B50] Jefferson G. (1984). “Transcript notation,” in Structures of Social Interaction: Studies in Conversation Analysis, eds J. Atkinson and J. Heritage (Cambridge: Cambridge University Press), 134–162.

[B51] Jones D. R. Mandell D. S. (2020). To address racial disparities in autism research, we must think globally, act locally. Autism 24, 1587–1589. 10.1177/1362361320948313 32998555

[B52] Kenny L. Hattersley C. Molins B. Buckley C. Povey C. Pellicano E. (2016). Which terms should be used to describe autism? Perspectives from the UK autism community. Autism 20, 442–462. 10.1177/1362361315588200 26134030

[B53] Kikuchi Y. Attaheri A. Wilson B. Rhone A. E. Nourski K. V. Gander P. E.. (2017). Sequence learning modulates neural responses and oscillatory coupling in human and monkey auditory cortex. PLoS Biol. 15:e2000219. 10.1371/journal.pbio.2000219 28441393 PMC5404755

[B54] Koegel R. Kim S. Koegel L. Schwartzman B. (2013). Improving socialization for high school students with ASD by using their preferred interests. J. Autism Dev. Disord. 43, 2121–2134. 10.1007/s10803-013-1765-3 23361918 PMC3672252

[B55] Koudenburg N. Postmes T. Gordijn E. H. (2017). Beyond content of conversation: the role of conversational form in the emergence and regulation of social structure. Personal. Soc. Psychol. Rev. 21, 50–71. 10.1177/1088868315626022 26874307

[B56] Lai M. C. Baron-Cohen S. (2015). Identifying the lost generation of adults with autism spectrum conditions. Lancet Psychiatry 2, 1013–1027. 10.1016/S2215-0366(15)00277-1 26544750

[B57] Leinonen E. Kerbel D. (1999). Relevance theory and pragmatic impairment. Int. J. Lang. Commun. Disord. 34, 367–390. 10.1080/136828299247342 10884907

[B58] Leinonen E. Ryder N. (2008). “Relevance theory and communication disorders,” in The Handbook of Clinical Linguistics, eds M. J. Ball, M. R. Perkins, N. Müller, and S. Howard (Blackwell: Cambridge), 49–60.

[B59] Loukusa S. Leinonen E. Kuusikko S. Jussila K. Mattila M. L. Ryder N.. (2007). Use of context in pragmatic language comprehension by children with Asperger syndrome or high-functioning autism. J. Autism Dev. Disord. 37, 1049–1059. 10.1007/s10803-006-0247-2 17072751

[B60] Mazurek M. O. (2014). Loneliness, friendship, and well-being in adults with autism spectrum disorders. Autism 18, 223–232. 10.1177/1362361312474121 24092838

[B61] McDonnell A. Milton D. (2014). “Going with the flow: reconsidering ‘repetitive behaviour' through the concept of ‘flow states',” in Good Autism Practice: Autism, Happiness and Wellbeing, eds G. Jones and E. Hurley (BILD: Birmingham), 38–47.

[B62] McGraw J. G. (1995). Loneliness, its nature and forms: an existential perspective. Man World. 28, 43–64. 10.1007/BF01278458

[B63] Millea M. (2013). “Paraphasia,” in Encyclopedia of Autism Spectrum Disorders, ed F. R. Volkmar (New York, NY: Springer).

[B64] Milton D. (2012). On the ontological status of autism: the double empathy problem. Disabil. Soc. 27, 883–887. 10.1080/09687599.2012.710008

[B65] Milton D. (2014). Embodied sociality and the conditioned relativism of dispositional diversity. Autonomy Crit. J. Interdiscipl. Autism Stud. 1, 1–7. Available online at: https://kar.kent.ac.uk/62632/

[B66] Milton D. Bracher M. (2013). Autistics speak but are they heard? J. BSA Medsoc Group 7, 61–69. Available online at: https://kar.kent.ac.uk/62635/

[B67] Milton D. Heasman B. Sheppard E. (2018). “Double empathy,” in Encyclopedia of Autism Spectrum Disorders, ed F. Volkmar (New York, NY: Springer). 10.1007/978-1-4614-6435-8_102273-1

[B68] Milton D. Timimi S. (2016). Does Autism Have an Essential Nature? Debate blog post. Available online at: https://kar.kent.ac.uk/62684/

[B69] Morrison K. E. DeBrabander K. M. Faso D. J. Sasson N. J. (2019a). Variability in first impressions of autistic adults made by neurotypical raters is driven more by characteristics of the rater than by characteristics of autistic adults. Autism 23, 1817–1829. 10.1177/1362361318824104 30848682

[B70] Morrison K. E. DeBrabander K. M. Jones D. R. Faso D. J. Ackerman R. A. Sasson N. J. (2019b). Outcomes of real-world social interaction for autistic adults paired with autistic compared to typically developing partners. Autism 24, 1067–1080. 10.1177/1362361319892701 31823656

[B71] Murray D. (2018). “Monotropism – an interest based account of autism,” in Encyclopedia of Autism Spectrum Disorders, ed F. Volkmar (New York, NY: Springer). 10.1007/978-1-4614-6435-8_102269-1

[B72] Murray D. (2020). “Monotropism – an interest based account of autism (second edition),” in Encyclopedia of Autism Spectrum Disorders, ed F. Volkmar (New York, NY: Springer). 10.1007/978-1-4614-6435-8_102269-2

[B73] Murray D. Lesser M. Lawson W. (2005). Attention, monotropism and the diagnostic criteria for autism. Autism 9, 139–156. 10.1177/1362361305051398 15857859

[B74] National Autistic Society (2018). Hidden Crisis: Autistic People Four Times More Likely to be Lonely Than General Public. Available online at: https://www.autism.org.uk/get-involved/media-centre/news/2018-04-25-hidden-crisis-autism-and-loneliness.aspx

[B75] Nelson A. A. Grahe J. E. Ramseyer F. (2016). Interacting in flow: an analysis of rapport-based behavior as optimal experience. SAGE Open 6:2158244016684173. 10.1177/2158244016684173

[B76] Ochs E. (1979). Transcription as theory. Dev. Pragmatics 10, 43–72.

[B77] Ochs E. Solomon O. (2010). Autistic sociality. Ethos 38, 69–92. 10.1111/j.1548-1352.2009.01082.x

[B78] Palinkas L. A. Horwitz S. M. Green C. A. Wisdom J. P. Duan N. Hoagwood K. (2015). Purposeful sampling for qualitative data collection and analysis in mixed method implementation research. Administration Policy Mental Health Mental Health Serv. Res. 42, 533–544. 10.1007/s10488-013-0528-y 24193818 PMC4012002

[B79] Papp S. (2006). A relevance-theoretic account of the development and deficits of theory of mind in normally developing children and individuals with autism. Theory Psychol. 16, 141–161. 10.1177/0959354306062532

[B80] Patton M. Q. (1999). Enhancing the quality and credibility of qualitative analysis. Health Serv. Res. 34, 1189–1208. 10591279 PMC1089059

[B81] Pearson A. Rees J. Rose K. Forster S. (2020). “This was just how this friendship worked”: Experiences of Interpersonal Victimisation in Autistic Adults. OSF Preprints. 10.31219/osf.io/amn6k PMC9645672 36605970

[B82] Pellicano E. Crane L. Gaudion K. the Shaping Autism Research Team (2017). Participatory Autism Research: A Starter Pack. London: UCL Institute of Education.

[B83] Pellicano L. (2014). A future made together: new directions in the ethics of autism research. J. Res. Special Educ. Needs 14, 200–204. 10.1111/1471-3802.12070_5

[B84] Peterson C. Wellman H. (2019). Longitudinal theory of mind (ToM) development from preschool to adolescence with and without ToM delay. Child Dev. 90, 1917–1934. 10.1111/cdev.13064 29660808

[B85] QSR International Pty Ltd (2020). NVivo (Released in March 2020). Available online at: https://www.qsrinternational.com/nvivo-qualitative-data-analysis-software/home

[B86] Rosqvist H. B. (2019). Doing things together: Exploring meanings of different forms of sociality among autistic people in an autistic work space. Alter 13, 168–178. 10.1016/j.alter.2019.03.003

[B87] Samson D. Apperly I. A. (2010). There is more to mind reading than having theory of mind concepts: new directions in theory of mind research. Infant Child Dev. 19, 443–454. 10.1002/icd.678

[B88] Sasson N. J. Faso D. J. Nugent J. Lovell S. Kennedy D. P. Grossman R. B. (2017). Neurotypical peers are less willing to interact with those with autism based on thin slice judgments. Sci. Rep. 7:40700. 10.1038/srep40700 28145411 PMC5286449

[B89] Schegloff E. A. (1992). Repair after next turn: the last structurally provided defense *(sic)* of intersubjectivity in conversation. Am. J. Sociol. 97, 1295–1345. 10.1086/229903

[B90] Sheppard E. Pillai D. Wong G. T. L. Ropar D. Mitchell P. (2016). How easy is it to read the minds of people with autism spectrum disorder? J. Autism Dev. Disord. 46, 1247–1254. 10.1007/s10803-015-2662-8 26603886

[B91] Sinclair J. (2010). Being autistic together. Disabil. Stud. Q. 30. 10.18061/dsq.v30i1.1075

[B92] Sirota K. G. (2010). Narratives of distinction: personal life narrative as a technology of the self in the everyday lives and relational worlds of children with autism. Ethos 38, 93–115. 10.1111/j.1548-1352.2009.01083.x

[B93] Solomon O. Bagatell N. (2010). Introduction: autism: rethinking the possibilities. Ethos 38, 1–7. 10.1111/j.1548-1352.2009.01078.x

[B94] Sperber D. Wilson D. (1986). Relevance: Communication and Cognition. Oxford: Blackwell Publishers.

[B95] Sperber D. Wilson D. (1995). Relevance: Communication and Cognition, 2nd Edn. Oxford: Wiley-Blackwell.

[B96] Sterponi L. de Kirby K. (2016). A multidimensional reappraisal of language in autism: insights from a discourse analytic study. J. Autism Dev. Disord. 46, 394–405. 10.1007/s10803-015-2679-z 26701673

[B97] Sterponi L. Fasulo A. (2010). “How to go on”: intersubjectivity and progressivity in the communication of a child with autism. Ethos 38, 116–142. 10.1111/j.1548-1352.2009.01084.x

[B98] Tager-Flusberg H. (1999). A psychological approach to understanding the social and language impairments in autism. Int. Rev. Psychiatry 11, 325–334. 10.1080/09540269974203 16467907 PMC1350917

[B99] Taylor I. Marrable T. (2011). Access to Social Care for Adults With Autistic Spectrum Conditions. London: Social Care Institute for Excellence and University of Sussex.

[B100] Timimi S. McCabe B. (2016). “What have we learned from the science of autism?,” in Re-thinking Autism: Diagnosis, Identity and Equality, eds K. Runswick-Cole, R. Mallett, and S. Timimi (London: Jessica Kingsley Publishers), 30–48.

[B101] Tracy S. J. (2010). Qualitative quality: eight “big-tent” criteria for excellent qualitative research. Qualitative Inquiry 16, 837–851. 10.1177/1077800410383121

[B102] Valtorta N. K. Kanaan M. Gilbody S. Ronzi S. Hanratty B. (2016). Loneliness and social isolation as risk factors for coronary heart disease and stroke: systematic review and meta-analysis of longitudinal observational studies. Heart 102, 1009–1016. 10.1136/heartjnl-2015-308790 27091846 PMC4941172

[B103] Verhoeff B. (2013). Autism in flux: a history of the concept from Leo Kanner to DSM-5. Hist. Psychiatry 24, 442–458. 10.1177/0957154X13500584 24573754

[B104] Warnell K. R. Redcay E. (2019). Minimal coherence among varied theory of mind measures in childhood and adulthood. Cognition 191, 1–10. 10.1016/j.cognition.2019.06.009 31229848

[B105] Watts S. (2014). User skills for qualitative analysis: Perspective, interpretation and the delivery of impact. Qual. Res. Psychol. 11, 1–14. 10.1080/14780887.2013.776156

[B106] Wearing C. (2010). Autism, metaphor and relevance theory. Mind Lang. 25, 196–216. 10.1111/j.1468-0017.2009.01386.x

[B107] Williams G. L. (2020). From anonymous subject to engaged stakeholder: enriching participant experience in autistic-language-use research. Res. All 4, 314–328. 10.14324/RFA.04.2.13

[B108] Wing L Gould J. (1979) Severe impairments of social interaction associated abnormalities in children: epidemiology classification. J. Autism Childh. Schizophr. 9, 11–29. 155684 10.1007/BF01531288

[B109] Wolk L. Edwards M. L. Brennan C. (2016). Phonological difficulties in children with autism: an overview. Speech Lang. Hear. 19, 121–129. 10.1080/2050571X.2015.1133488

[B110] Wood R. (2019). Autism, intense interests and support in school: from wasted efforts to shared understandings. Educ. Rev. 73, 1–21. 10.1080/00131911.2019.1566213

[B111] Woods R. Milton D. Arnold L. Graby S. (2018). Redefining critical autism studies: a more inclusive interpretation. Disabil. Soc. 32, 974–979. 10.1080/09687599.2018.1454380

