# Supplementary material for: Mutual (Mis)understanding: Reframing Autistic Pragmatic “Impairments” Using Relevance Theory
Source: Front Psychol. 2021 Apr 29;12:616664. doi: 10.3389/fpsyg.2021.616664 (PMC8117104; doi:10.3389/fpsyg.2021.616664)
Supplement: Supplementary file 1 [file Data_Sheet_1.PDF]

Stage One: Minimal conversation prompts

(Each pair will be provided with a strip of paper with two prompts on and advised that these prompts are just to guide.)

What does loneliness mean to you?

Do results of Loneliness Experiment (*provided*) surprise you?

What is your experience of loneliness in the Brighton and Hove area?

What do you think could be done in the city to address loneliness?

What could be done in Brighton and Hove to combat loneliness?

Do you think loneliness is a problem in Brighton and Hove?

What could be done to encourage strangers to talk together more?
