# Supplementary material for: Mutual (Mis)understanding: Reframing Autistic Pragmatic “Impairments” Using Relevance Theory
Source: Front Psychol. 2021 Apr 29;12:616664. doi: 10.3389/fpsyg.2021.616664 (PMC8117104; doi:10.3389/fpsyg.2021.616664)
Supplement: Supplementary file 2 [file Data_Sheet_2.PDF]

Motifs and codes

flow

<u>presence of flow</u>

- fast-pace enthusiasm (+ short turns)
  - special interest enthusing (e.g. dogs)

<u>stilted / lacking flow</u>

- gaps, lapses
- overlapping turns: cross-talk: [see **tuning in**]
- long turns / monologues:
  - speaker dominating / holding the floor
  - listener allowing speaker 'to go on…' (giving space where speaker's speech is laboured)[1]
- interruptions
- non / minimally / only tangentially relevant responses

tuning-in

- checking Qs
  - have I understood you? → *absence of mutuality / effortful*
  - have I been clear/ do you understand me? → *absence of mutuality / effortful*
  - invitation to build 'we-ness' ('*You know?*') → *presence of mutuality / building*
- overlapping turns
  - finish each other's sentences → *attuned*
  - cross-talk → *not attuned* [see **flow**]
- rapport / affect

---

[1] *Several **A**s appear to have laboured speech at times.*

- mirroring / echoing
  - specific words / phrases
  - ideas / parallel situations / anecdotes
- accommodation /converging
- extra ‘efforts’
- jokes/humour:
  - deflective of emotional content → *moving away*
  - creating we-ness / affect / bonding / sharing → *moving together*

running along the edges of meaning

- left-field topic development
- abrupt topic changes / ‘low demand for coherence’
- non-words / onomatopoeia etc.

mutual manifestness

- moments where it was expected but is not there
- moments where it is there- things don’t need to be said
