# Supplementary material for: Mutual (Mis)understanding: Reframing Autistic Pragmatic “Impairments” Using Relevance Theory
Source: Front Psychol. 2021 Apr 29;12:616664. doi: 10.3389/fpsyg.2021.616664 (PMC8117104; doi:10.3389/fpsyg.2021.616664)
Supplement: Supplementary file 3 [file Data_Sheet_3.PDF]

# Transcription Conventions

| | |
|---|---|
| . | Period indicates a falling, or final, intonation contour, not necessarily the end of a sentence. |
| ? | Question mark indicates rising intonation, not necessarily a question. |
| , | Comma indicates “continuing” intonation, not necessarily a clause boundary. |
| ↑↓ | Upward and downward pointing arrows indicate marked rising and falling shifts in intonation (arrows come before the syllable in question) |
| ::: | Colons indicate stretching of the preceding sound, proportional to the number of colons |
| - | A hyphen after a word or a part of a word indicates a cut-off or self-interruption with level pitch |
| word | Underlining indicates stress or emphasis. |
| WOrd | Upper case indicates loudness. |
| °word° | Degree signs enclose whispered speech |
| £word£ | Pound sign indicates smiley voice, or suppressed laughter |
| = | Equal sign indicate no break or delay between the words thereby connected. |
| <word> | Indicates slowed down delivery relative to surrounding talk |
| >word< | Indicates speeded up delivery relative to surrounding talk |
| (()) | Double parentheses enclose descriptions of conduct. |
| (word) | When all or part of an utterance is in parentheses, this indicates uncertainty on the transcriber’s part. |
| () | Empty parentheses indicate that something is being said, but no hearing can be achieved. |
| (1.2) | Numbers in parentheses indicate silence in tenths of a second. |
| (.) | A dot in parentheses indicated a “micropause,” hearable but not readily measurable. |
| [ | Separate left square brackets, one above the other on two successive lines with utterances by different speakers, indicates a point of overlap onset. |
| ] | Separate right square brackets, one above the other on two successive lines with utterances by different speakers, indicates a point of overlap ending. |
| … | Ellipsis |
| (-) | Indicates unintelligible speech, each dash pertains to a syllable. |
